# IKAROS Deletions Dictate a Unique Gene Expression Signature in Patients with Adult B-Cell Acute Lymphoblastic Leukemia

**DOI:** 10.1371/journal.pone.0040934

**Published:** 2012-07-25

**Authors:** Ilaria Iacobucci, Nunzio Iraci, Monica Messina, Annalisa Lonetti, Sabina Chiaretti, Emanuele Valli, Anna Ferrari, Cristina Papayannidis, Francesca Paoloni, Antonella Vitale, Clelia Tiziana Storlazzi, Emanuela Ottaviani, Viviana Guadagnuolo, Sandra Durante, Marco Vignetti, Simona Soverini, Fabrizio Pane, Robin Foà, Michele Baccarani, Markus Müschen, Giovanni Perini, Giovanni Martinelli

**Affiliations:** 1 Department of Hematology and Oncological Sciences “L. and A. Seràgnoli”, S. Orsola Malpighi Hospital, University of Bologna, Bologna, Italy; 2 Department of Biology, University of Bologna, Bologna, Italy; 3 Division of Hematology, Department of Cellular Biotechnologies and Hematology, “Sapienza” University of Rome, Rome, Italy; 4 Cellular Signalling Laboratory, Department of Human Anatomy, University of Bologna, Bologna, Italy; 5 Gruppo Italiano Malattie Ematologiche Maligne dell’Adulto (GIMEMA) Data Center, GIMEMA Foundation, Rome, Italy; 6 Department of Genetics and Microbiology, University of Bari, Bari, Italy; 7 CEINGE Biotecnologie Avanzate and Department of Biochemistry and Medical Biotechnology, University of Naples Federico II, Naples, Italy; 8 Leukemia Research Program, Childrens Hospital Los Angeles, University of Southern California, Los Angeles, California, United States of America; Clinica Universidad de Navarra, Spain

## Abstract

**Background:**

Deletions of IKAROS (*IKZF1*) frequently occur in B-cell precursor acute lymphoblastic leukemia (B-ALL) but the mechanisms by which they influence pathogenesis are unclear. To address this issue, a cohort of 144 adult B-ALL patients (106 *BCR-ABL1*-positive and 38 B-ALL negative for known molecular rearrangements) was screened for *IKZF1* deletions by single nucleotide polymorphism (SNP) arrays; a sub-cohort of these patients (44%) was then analyzed for gene expression profiling.

**Principal Findings:**

Total or partial deletions of *IKZF1* were more frequent in *BCR-ABL1*-positive than in *BCR-ABL1*-negative B-ALL cases (75% *vs* 58%, respectively, p = 0.04). Comparison of the gene expression signatures of patients carrying *IKZF1* deletion *vs* those without showed a unique signature featured by down-regulation of B-cell lineage and DNA repair genes and up-regulation of genes involved in cell cycle, *JAK-STAT* signalling and stem cell self-renewal. Through chromatin immunoprecipitation and luciferase reporter assays we corroborated these findings both *in vivo* and *in vitro*, showing that Ikaros deleted isoforms lacked the ability to directly regulate a large group of the genes in the signature, such as *IGLL1*, *BLK*, *EBF1*, *MSH2, BUB3, ETV6, YES1, CDKN1A* (p21), *CDKN2C* (p18) and *MCL1*.

**Conclusions:**

Here we identified and validated for the first time molecular pathways specifically controlled by *IKZF1*, shedding light into *IKZF1* role in B-ALL pathogenesis.

## Introduction

Based on cytogenetic and molecular analyses, B-cell precursor acute lymphoblastic leukemia (B-ALL) carries hyperdiploidy, hypodiploidy, translocations t(12;21)[*ETV6-RUNX1*], t(1;19)[*TCF3-PBX1*], t(9;22)[*BCR-ABL1*] and rearrangement of the *MLL* gene. The frequencies of these abnormalities vary among children and adults and their detection is important in diagnosis and risk stratification [1], [2]. In addition, approximately one quarter of B-ALL cases, defined as “B-NEG” throughout the text, lack recurring cytogenetic and molecular abnormalities, and the genetic basis of these cases is unknown [1], [3], [4]. Recently, genome-wide approaches to study DNA copy number abnormalities and loss of heterozygosity (LOH) events led to impressive progresses and revolutionized the knowledge of the genetic basis of ALL [5], [6], [7], [8]. In B-ALL a high frequency of genetic abnormalities in key pathways, including lymphoid differentiation, cell cycle regulation, tumor suppression, and drug responsiveness, have been identified [9], [10]. Among these abnormalities, deletions of *IKZF1* gene, encoding the transcription factor Ikaros, were found in about 28% of pediatric *BCR-ABL1* negative ALL cases and in more than 80% of adult *BCR-ABL1* positive cases [7], [11]. Ikaros is required for the earliest stages of lymphoid lineage commitment and for tumor suppression in mouse [12], [13], [14]. In many cases, *IKZF1* deletions lead to the expression of dominant-negative Ikaros splice variants (e.g. Ik6) that are characterized by loss of N-terminal zinc finger DNA binding domains, and retention of the C-terminal dimerization domain [7], [11]. *IKZF1* deletions were significantly associated with an increased relapse rate and adverse events and were correlated with poor outcome in both pediatric and adult patients with *BCR-ABL1*-positive ALL [15], [16], [17]. However, the precise mechanism(s) by which Ikaros alterations contribute to leukemogenesis and worse prognosis have not yet been clearly defined. To investigate this aspect, we correlated the genomic status of the *IKZF1* gene in a cohort of adult B-ALL patients (*BCR-ABL1* positive and B-ALL negative for known molecular rearrangements, here named B-NEG, cases) with transcription profiles. Furthermore, through chromatin immunoprecipitation and luciferase assays, we have defined how full-length and deleted forms of *IKZF1* can affect gene expression. Our results show that independently from *BCR-ABL1*, the loss of *IKZF1* function dictates a unique expression signature characterized by down-regulation of B-cell lineage and DNA repair genes and up-regulation of genes involved in cell-cycle progression, JAK/STAT signalling and stem cell self-renewal, shedding light on the molecular mechanisms that may underlie the leukemogenic potential of Ikaros deletions.

## Results

### *IKZF1* Deletions Occur in 71% of B-precursor Adult ALL (B-NEG and *BCR-ABL1*+)

**Figure 1:**
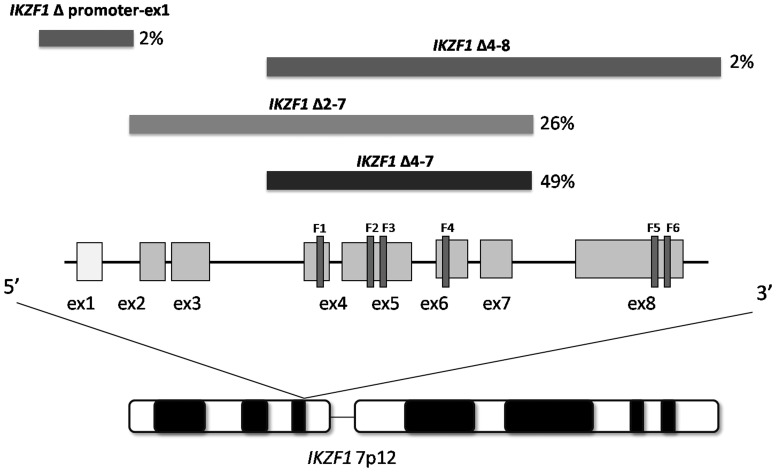
*IKZF1* deletion patterns in adult ALL patients. Schematic representation of the *IKZF1* gene on chromosome 7p12, with coding exons (ex) in gray and the first non-coding exon in white. N-terminal zinc-fingers (F1–F4) show DNA-binding activity and C-terminal ones (F5–F6) mediate dimerization of the protein. Black segment reports indicate the genomic extent of the *IKZF1* deletions of the most common deletions. Percentage is reported for each type of deletion.

SNP arrays analyses revealed that among the 144 patients tested (**Table 1**), deletions of *IKZF1* occurred in 22 out of 38 (58%) B-NEG ALL cases and in 80 out of 106 (75%) adult *BCR-ABL1*+ cases, indicating that *IKZF1* deletion is more frequent in the *BCR-ABL1*+ ALL subgroup (p = 0.04). When the whole cohort of adult B- ALL patients (B-NEG and *BCR-ABL1*+) was considered, *IKZF1* deletion occurred in 102 (71%) cases. Deletions were predominantly mono-allelic (89% *vs* 11%) and showed a variable pattern of extension over the *IKZF1* locus (**Figure 1**). Among patients carrying deletions in *IKZF1*, 19 (19%) showed a complete deletion of the locus, whereas in 83 (81%) patients only a subgroup of exons or the genomic region immediately upstream of *IKZF1* was deleted. In particular, in 50 patients (49%) there was a deletion of the coding exons 4 through 7, causing expression of the dominant-negative isoform Ik6, lacking the DNA binding domain. In 27 cases (26%) we identified the loss of exons 2 through 7, producing an Ikaros isoform lacking the translation start site. In the remaining cases, the deletion either included exon 8 or the promoter and exon 1 regions, contributing to *IKZF1* haploinsufficiency. We did not observe differences in the occurrence of different types of deletions across *BCR-ABL1*+ and B-NEG cases.

**Table 1:** Demographics and Clinical Characteristics of patients with B-progenitor positive Acute Lymphoblastic Leukemia.

Patient Characteristics	*BCR-ABL1*-positive ALL	B*-*NEG ALL
Number	106	38
Age (yrs): median (range)	53 (18–76)	31 (16–54)
Blast (%): median (range)	90 (18–99)	95 (80–100)
Sex: Male (%)/Female (%)	63 (59)/43 (41)	27 (71)/11(29)
Leukocytes/µl: median (range)	23.4 (1.4–302.0)	43 (3–357)

### Gene Expression Analysis Revealed a Unique Signature Associated with *IKZF1* Deletions

**Figure 2:**
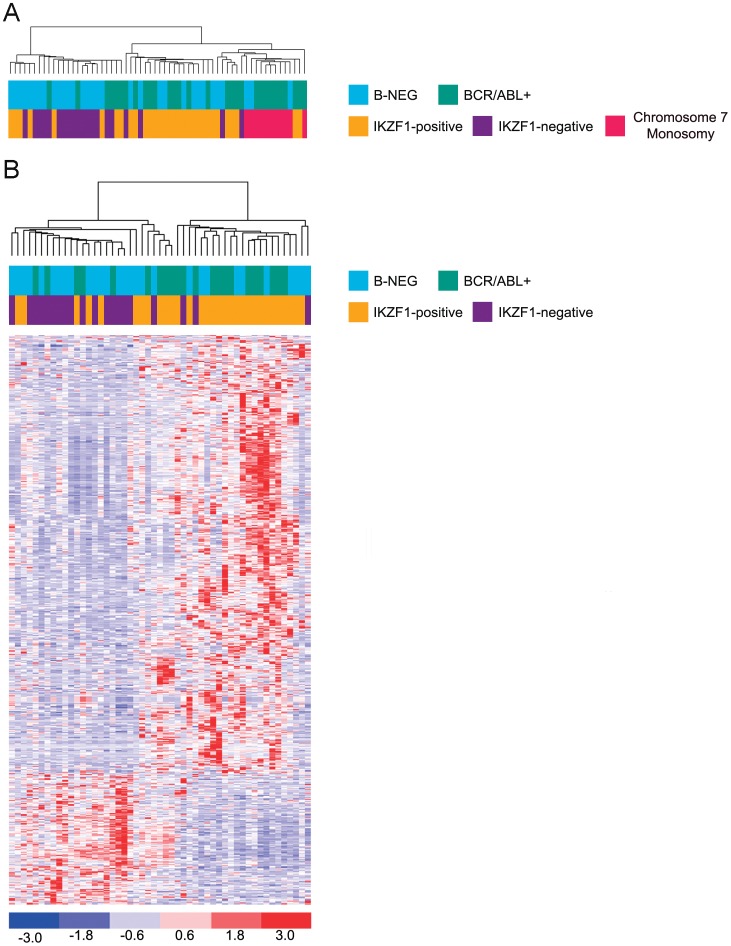
Gene expression profile in B-ALL patients. A) Hierarchical clustering of 62 B-ALL cases based on 1602 probes resulting from ANOVA. Cases with chromosome 7 monosomy branched together in a unique homogeneous subcluster which was included in a cluster comprising the majority of *IKZF1*-deletion positive samples; B) Gene expression profile of 51 B-ALL adult samples (excluding chromosome 7 monosomy cases), based on 684 probes identified by t-test. Each row represents a probe set, each column represents a single ALL sample. Bottom, color scale indicating the relative levels of expression: dark blue, the lowest levels of expression; red, highest levels of expression.

To evaluate whether cases harbouring *IKZF1* alterations display a peculiar gene expression profile, we performed two supervised analyses on gene expression data from 62 B-ALL patients (30 *BCR-ABL+* and 32 B-NEG ALL). *IKZF1* deletion was equally distributed in this experimental setting among B-ALL subtypes with a rate of 53% and 47% in *BCR-ABL1+* and B-NEG ALL, respectively (**Table S1**). Since some cases (8 *BCR-ABL1*+ and 3 B-NEG) lost *IKZF1* as the result of the monosomy of chromosome 7, where *IKZF1* is located (7p12), we decided to divide our cohort in 3 groups: A) cases with *IKZF1*-deletions (n = 31), B) cases without *IKZF1* deletions (n = 20) and C) cases with loss of *IKZF1* due to monosomy 7 (n = 11). Gene expression profiles of these three groups were compared by Analysis of Variance (ANOVA). Results showed that 1602 probes, corresponding to 1130 genes, are differentially expressed and grouped patients in three sub-clusters with distinct expression profiles. It should be noted that one sub-cluster was constituted by almost all cases (10 out of 11) with monosomy of chromosome 7 (**Figure 2A**): this result depended on the fact that 84.6% of down-modulated genes are indeed located on chromosome 7, thus complicating and probably masking the genuine expression signature mediated by *IKZF1* alone. Notably, a clear clustering of monosomy 7 cases was also evident by unsupervised clustering (data not shown). Therefore, to overcome this issue, we specifically focused on patients with or without deletions in the *IKZF1 locus*. By t-test analysis 684 probes, corresponding to 422 genes, were found differentially expressed between *IKZF1*-deletion positive and negative patients: 128 genes were down-modulated and 294 genes up-regulated in *IKZF1*-deletion positive (median FDR: 13%) (**Tables S2 and S3**, respectively) (**Figure 2B**).

### Deletions of Ikaros Deregulate Multiple Genes Involved in Specific B-cell Intracellular Pathways

To identify peculiar critical pathways affected by Ikaros deletions, the 294 up-regulated and 128 down-regulated genes were analyzed in terms of biological function using the Metacore pathway-mapping software and the DAVID software. When considering the top 10 statistically down-regulated networks (**Figure S1A**), we detected several genes involved in B-cell differentiation pathway (*BLK*, *BTK*, *IGLL1, CD22, PLCG2, MAP3K1, VPREB1*) and, to a lesser extent, in apoptosis and cell cycle (*BUB3, BID, 14-3-3 protein beta/alpha*). Functional analysis, performed using the DAVID software, confirmed these findings and, in addition, highlighted the DNA repair gene category (*MSH2, MSH6, UBE2V2*) as significantly down-modulated.

Among the up-regulated genes, a substantial number appeared to be involved in cell cycle and apoptosis regulation (*STK17B, SERPINB9, CDKN1A (p21), CDKN2C (p18), FLT1, LATS2, JUND, MCL1, etc*). Importantly, the *JAK-STAT* signalling pathway (*YES1*, *CISH, SOCS1, SOCS3, SOCS5, STAT3*), DNA damage (*GADD45A, GADD45B, REL*), hematopoietic stem cell (HSC)-affiliated genes (*CDKN1A*, *SOCS2*, *SOCS3*, *ALDH2, ETV6* and *MLLT3)* (**Figure S1B**) as well as the early primed erythroid factor *KLF9* and the late myeloid gene *ID2,* indicating their premature induction, were also found up-regulated.

Furthermore, we also evaluated the expression of a set of genes previously shown to play a role in leukemogenesis, namely, *EBF1, CD200, PAX5, RB1, CDKN2A, LEF1, BTG1* and *RAG* [6]. This analysis highlighted an up-regulation of *CD200* in *IKZF1*-deleted patients and a down-modulation of *RAG* and *EBF1* (data not shown). This is in agreement with previous data showing that Ikaros regulates IGHV recombination [18].

**Figure 3:**
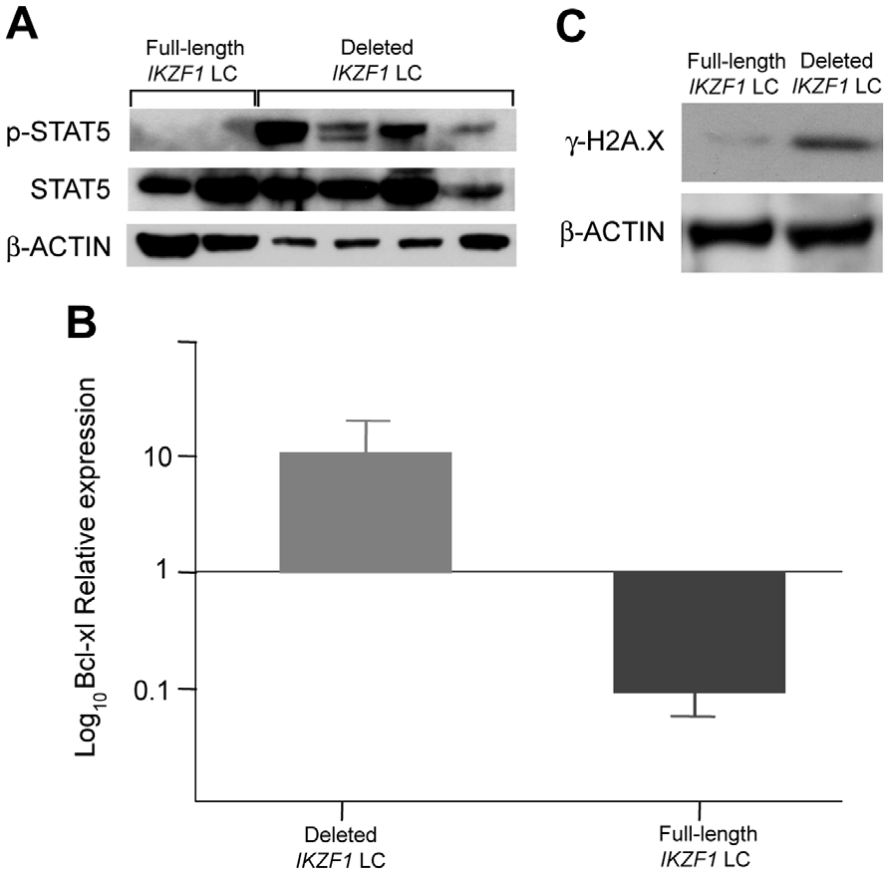
JAK-STAT5 signalling in Ikaros-rearranged ALL. Western Blot analysis of STAT5 in *IKZF1* deleted and wild-type leukemia primary cells (A) showed an increased phosphorylation of STAT5 in cells with Ikaros deletion. This was correlated with an increased production of Bcl-xl (B) as determined by RQ-PCR. (C) Western Blot analysis showed a marked increased phosphorylation of histone H2A.X in leukemia cells with Ikaros deletion compared to normal *IKZF1* cells. Abbreviations: LC indicates leukemia cells. β-ACTIN was used as a loading control of the WB.

To understand whether *IKZF1* deletions may affect intracellular signalling we compared the activation status of the *JAK-STAT* pathway between primary leukemia cells obtained from four patients with, and two without, *IKZF1* deletion. As previously demonstrated in murine pro-B lymphocytes [19], the expression of isoforms derived from deletion was associated with increased phosphorylation of STAT5 (**Figure 3A**) and significantly induced mRNA expression of the downstream anti-apoptotic Bcl-xl (**Figure 3B**). Since in children with B-ALL and *IKZF1* deletions, *JAK2* mutations have been reported to constitutively activate *JAK2* [20], we performed a mutational screening in order to assess whether this was also the case in our cohort. Overall 17 *BCR-ABL1*-positive and 16 B-NEG negative ALL cases were analyzed for *JAK2* mutations. These cases included six patients on which western blot analysis for pSTAT5 was performed. No mutations were found, except for the silent D820 and L830 mutations identified each in single cases and for the missense E444K and D489N, identified both in one *BCR-ABL1* negative patient. The latter mutations were outside the pseudokinase domain of *JAK2*.

Moreover, to investigate whether Ikaros deletions correlate with accumulation of DNA damage, as suggested by gene expression signature, we examined the phosphorylation of histone H2A.X (γ-H2A.X) as an indicator of the cellular response to DNA damage [21]. Primary leukemia cells with Ikaros deletion showed a marked increase in the basal phosphorylation of H2A.X when compared to leukemia cells with wild-type Ikaros, suggesting that Ikaros deletions co-exist in cells with the propensity to genome instability (**Figure 3C**).

### *IKZF1* Deleted Isoforms Lack the Ability to Directly Regulate a Large Group of Target Genes *in vivo*

Thereafter, to determine whether *IKZF1* is directly involved in the regulation of putative target genes identified in gene expression analysis, the promoter regions mapping around their transcriptional start sites were scanned for the presence of the conserved *IKZF1* binding site (GGGAA) [22]. We found several potential Ikaros binding sites in the promoters of many deregulated genes, such as *IGLL1*, *BLK*, *EBF1, BUB3, MSH2, MSH6*, *BTK*, *VPREB1*, *CD22*, *MCL1, ETV6*, *YES1, CDKN1A* (p21) and *CDKN2C* (p18). Assessment of direct association of Ikaros isoforms to tested promoters was determined by applying cross-linking chromatin immunoprecipitation (ChIP) assay to two different cell lines: SD-1 and BV-173 expressing *IKZF1* full-length protein or the IK6 deleted isoform, respectively [11]. A pre-immune serum was used as a negative control to determine the baseline of the nonspecific background.

**Figure 4:**
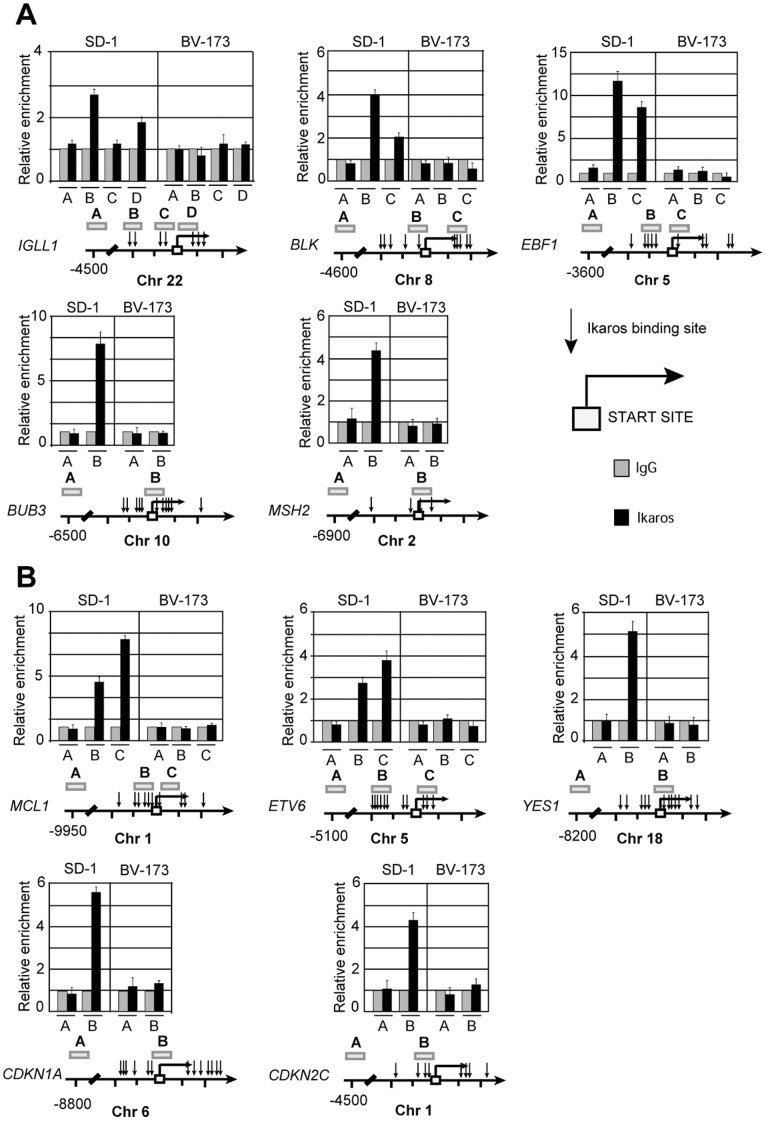
The Ik6 isoform lacks the ability to regulate directly a large group of target genes *in vivo*. Analysis of down-regulated (A) and up-regulated (B) genes in *IKZF1*-deletion positive cases through chromatin Immunoprecipitation assay (ChIP). The experiments were performed in SD-1 and in BV-173 cell lines, expressing full-length Ikaros transcription factor and deleted isoform IK6, respectively. Results represent the average of three independent experiments in which each region was amplified by qPCR in triplicate. Standard error is indicated. Promoter diagram: bent arrow, transcription start site; black arrow, Ikaros binding sites; open boxes, amplicons indicated with a capital letter; chromosome and coordinates (bp) are also given.

As shown in **Figure 4**, the promoter regions of the selected genes, such as *IGLL1*, *BLK*, *EBF1*, *BUB3, MSH2, MCL1, ETV6, YES1, CDKN1A* (p21) and *CDKN2C* (p18) were bound *in vivo* only by *IKZF1* full-length protein in SD-1 cell line, but not by Ik6 mutant in BV-173 cells. A distal DNA region (Amplicon A) for each gene, lacking *IKZF1* consensus sites, was tested in ChIP as a negative control.

Although transcriptionally affected by *IKZF1* deletions, the promoters of *MSH6, BTK, VPREB1* and *CD22,* and genes were not bound by *IKZF1*, neither in SD-1 nor in BV-173 cell lines (**Figure S2**), thence suggesting that these genes may be “secondary targets” of Ikaros transcriptional activity. A similar result was also obtained when ChIP was applied to primary cells of two B-ALL patients: one carrying a full-length *IKZF1* and the second one carrying the deleted Ik6 isoform (**Figure S3**).

**Figure 5:**
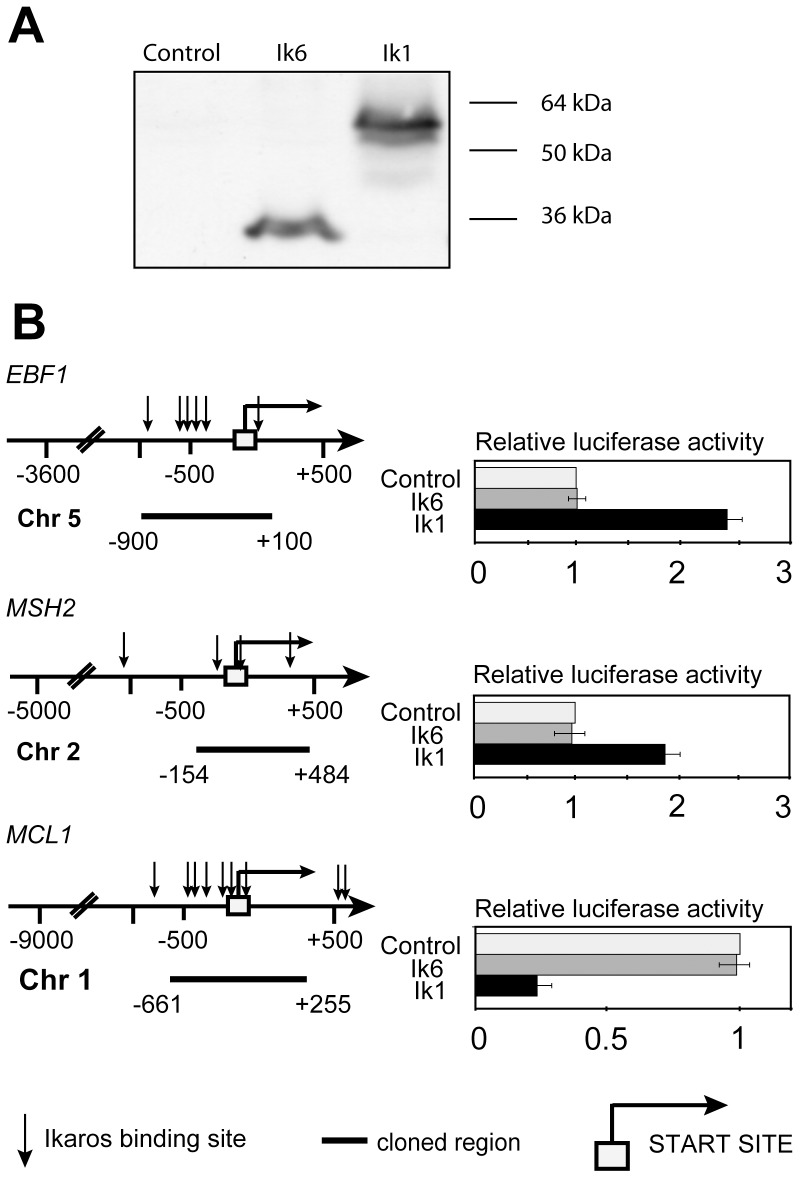
The Ik6 isoform cannot regulate transcription of the *EBF1, MSH2, and MCL1* promoters in a transient luciferase assay. A) Western blotting of Ik1 and Ik6 proteins transiently expressed by respective vectors when transfected into HEK-293 cells. B) Left: schematic representation of the promoters cloned upstream of the pGL3basic-Luciferase vector. Right: relative luciferase activity of the reporters determined by co-transfection with either an Ik6 or an Ik1 expression vector as compared to that obtained with the empty vector which was arbitrarily set to 1. Results represent the average of three independent experiments.

To confirm that Ik1 full-length is required to activate or repress transcription, we performed experiments using Luciferase reporter constructs carrying the promoter regions of three different genes illustrative of those whose promoters were bound by Ik1 in ChIP experiments. Specifically, we chose to analyze *EBF1* and *MSH2* which are down-regulated in *IKZF1*-deletion positive cases and *MCL1* which is up-regulated. Recombinant reporters [*EBF1*(−900/+100); *MSH2*(−154/+484); *MCL1*(−661/+255)] were transiently co-transfected in HEK-293 cells along with either a Ik6 or a Ik1 expression vector and luciferase activity was quantified. Expression levels of the Ik1 and Ik6 exogenous proteins in transfected cells was quantified by Western Blotting (**Figure 5A**).

As expected, results show that the *IKZF1* full-length protein can both positively (*EBF1* and *MSH2*) and negatively (*MCL1*) modulate the luciferase activity of the luc-reporters, whereas the deleted Ik6 isoform cannot (**Figure 5B**).

Overall, these data support a model in which Ik6 has lost the ability to bind target genes and to properly control their transcription profile.

## Discussion

While different studies have established that Ikaros alterations have a pivotal role in the leukemogenesis of *BCR-ABL1* positive and negative ALL [7], [11], [17], the precise mechanisms by which they promote leukemogenesis or cooperate with known molecular rearrangements (e.g. *BCR-ABL1*) remain to be elucidated. Here, we took advantage of high-resolution SNP array, transcriptional profiling and chromatin immunoprecipitation approaches to explore these mechanisms. In physiologic conditions, Ikaros is expressed in multipotent, self-renewing hematopoietic stem cells (HSCs) as well as in their committed progenitors and its expression increases during lymphocytes development [23], [24]. In mice, heterozygous disruption of Ikaros leads to development of T-cell malignancies [12], [25], [26], whereas homozygous inactivation is responsible for severe lymphoid cell defects with abnormal thymopoiesis and perturbed myelopoeisis [12] [14], [27], [28]. Recently [29], Ikaros was described as a key coordinator of the lymphoid-myeloid genetic network that is responsible for activation of lymphoid signatures in the HSCs and for repression of lineage inappropriate signatures downstream of HSCs. Perturbations of this tightly regulated network may clearly alter hematopoiesis development and promote leukemogenesis.

Collectively, in the largest cohort of adult B-ALL patients (106 *BCR-ABL1* positive and 38 B-NEG) reported so far we detected deletions of *IKZF1* gene in 71% B-ALL cases with a variable pattern of breakpoints. In the majority of cases, alterations were found to encode transcripts lacking the DNA binding domain acting as dominant negative isoforms, or transcripts lacking the translation start site contributing to Ikaros haploinsufficiency.

We further investigated the effects of these alterations, identifying a unique gene expression profile for patients with Ikaros deletions unrelated to *BCR-ABL1* rearrangement but specifically associated with Ikaros genomic status. This signature was characterized by down-regulation of genes required for B-cell lineage development and DNA repair upon response to DNA damage and up-regulation of genes involved in cell cycle/apoptosis, JAK-STAT signalling and stem cell self-renewal. Although our data were only partially consistent with those reported by Mullighan et al. in children with high-risk ALL [17] and with recent gene expression profiling studies that have identified the new *BCR-ABL1*-like subtype [30], here for the first time we showed by ChIP analysis that Ikaros can bind *in vivo* the promoters of many genes differentially expressed between *IKZF1* deletion and *IKZF1* wild-type cases. Among these, we formally demonstrated, through luciferase reporter assay, that *EBF1*, *MSH2* and *MCL1*, involved in B-cell development, DNA repair and inhibition of apoptosis, respectively, are novel identified Ikaros targets. *EBF1* is a chief determinant of B cell fate, inducing expression of B cell–specific genes and *IGH* gene recombination and also represses the myeloid developmental program [31]. In *IKZF1* ^−/−^ multipotent progenitors, *EBF1* ectopic expression was shown to rescue B-cell development and redirect, at low frequency, myeloid progenitors toward the B-cell lineage. This successful ‘rescue’ suggested that Ikaros may be required for the developmental induction of *EBF1* but this hypothesis was thereafter not addressed [18]. Here, beyond demonstrating that *EBF1* is an Ikaros target, we also provided strong lines of evidence that Ikaros deletion directly reduces activation of *EBF1* in leukemia cells contributing to an extensive block of B-cell differentiation.

We also demonstrated that Ikaros can bind the promoter region of *MSH2* indicating a novel role for Ikaros as a direct regulator of DNA repair. Ikaros is expressed in multipotent, self-renewing HSC [23] in which the repair of potential DNA damage is crucial to avoid stem cell depletion. Reese and colleagues showed that the HSC of MSH2 null mice possess an enhanced capacity to survive the exposure to the methylating agent, temozolomide (TMZ), a compound that causes DNA damage requiring mismatch repair mechanisms [32]. Although additional experiments will be required to formally demonstrate the role of Ikaros in maintaining genomic integrity, the accumulation of DNA damage we found in cells with Ikaros deletions, together with our ChIP/luciferase results, lead us to hypothesize that loss of Ikaros could contribute to increase genomic instability in leukemia stem cells. Moreover, the up-regulation of genes involved in inhibition of apoptosis (e.g. *MCL1*) may promote the survival of leukemia cells with accumulated DNA damage and their expansion as suggested by an up-regulation of genes involved in cell cycle progression (*AHR, AHR, TUBB2A, RAPGEF3, SERTAD1, FLT1*). Noteworthy, among the genes up-regulated we found an over-expression of *CDKN1A* (p21) which has been recently [33] demonstrated to be indispensable for maintaining self-renewal of leukemia stem cells and avoiding their exhaustion. Based on our findings from SNP array, gene expression profile and ChIP/luciferase results we propose the following model: in physiologic condition Ikaros is expressed in the multipotent, self-renewing hematopoietic stem cells [34] where it is responsible for i) activation of a cascade of lymphoid genes, allowing lineage differentiation; ii) tight control of cell cycle progression and iii) DNA repair. Downstream of the hematopoietic stem cells, Ikaros restricts the multilineage potential of lymphoid and myeloid progenitors by repressing lineage-inappropriate genetic programs including hematopoietic stem cell–specific genes. Deletions result in the majority of cases in the production of isoforms unable to bind the DNA, which determine a block of differentiation, increased proliferation [35], continuous self-renewal and accumulation of DNA damage.

In conclusion, our findings shed light on a new subgroup of B-ALL, including *BCR-ABL1* positive and B-NEG patients, characterized by a unique signature dependent on Ikaros deletion. The prognostic impact of Ikaros deletions, that translates in an increased relapse rate and an overall poor outcome in both *BCR-ABL1* positive patients and *BCR-ABL1* negative patients (**Tables S4 and S5**) [15], [16], [17], [36], [37] highlights the importance to perform a screening of deletions at diagnosis. Finally, a comprehensive understanding of the molecular consequences of Ikaros deletion may help in identifying novel treatment options for B-cell–progenitor ALL patients.

## Materials and Methods

### Patients

This study was approved by GIMEMA AL Working Party (Gruppo Italiano Malattie EMatologiche Maligne dell’Adulto Acute Leukemia). All patients gave informed consent to blood collection and biologic analyses, in agreement with the Declaration of Helsinki. Overall, we collected peripheral blood or bone marrow samples from a cohort of 144 newly diagnosed adult ALL patients (106 *BCR-ABL1*-positive, 38 negative for the major molecular rearrangements, B-NEG) enrolled from 1996 to 2008 in different clinical trials of GIMEMA AL Working Party (LAL 0496, LAL 2000, LAL 0201-B and LAL 1205) and institutional protocols. Detailed information on the clinical trials of GIMEMA AL Working Party is reported in the **Table S6**. The main clinical and biological characteristics of the patients are shown in **Table 1**. The human *BCR-ABL1-*positive leukemia cell lines SD1 and BV-173 were also included in the analysis. They were obtained from DMSZ (Deutshe Sammlung von Mikroorganismen und Zellkulturen GmbH, Braunschweig, Germany) and maintained in culture following the DMSZ recommendations.

### Single Nucleotide Polymorphism (SNP) Array Analysis

Samples were genotyped with GeneChip® Human Mapping 250 K NspI and Genome-Wide Human SNP 6.0 arrays (Affymetrix) according to the manufacturer’s instructions and as previously described [11], [38]. Copy number aberrations in *IKZF1* were scored using the Hidden Markov Model and the segmentation approach available within the Partek Genomic Suite (Partek Inc) software package as well as by visual inspection. Copy number aberrations were also analyzed and eventually confirmed using Genotyping Console 3.0 (Affymetrix). SNP array data have been previously deposited according to [11].

### *IKZF1* Genomic Quantitative and Qualitative PCR

In order to confirm the *IKZF1* deletions detected by SNP array and to define the extension of alterations, *IKZF1* genomic quantitative PCR (Q-PCR) of all *IKZF1* coding exons was performed as described by Mullighan et al. [7] using a 7900 Real-Time PCR system and 7900 System Software (Applied Biosystems). Characterization of breakpoints occurring in *IKZF1* was carried out by long-range PCR experiments, as previously described [11].

### RNA Extraction and Gene Expression Profiling Experiments

Sixty-two, including 32 B-NEG and 30 *BCR-ABL1+* B-ALL, out of 144 samples analyzed by SNP, were also studied for gene expression profiling (44%). HGU133 Plus 2.0 gene chips (Affymetrix) were used to perform gene expression profile experiments. Thawed or freshly isolated cells were homogenized and total RNA was extracted using the RNeasy mini kit (Qiagen). The quality of total RNA was checked by agarose gel electrophoresis and RNA concentration was determined by measuring the absorbance at 260 nm; for all samples, the 260/280 ratio was >1.8, as required for microarray analysis. Array preparation was performed as described [39]. Gene expression data have been previously deposited in the context of the MILe Study [40] with GEO accession number GSE13204.

### Gene Expression Data Analysis

Oligonucleotide microarray and gene expression data analysis were performed using the dChip software (www.dchip.org) [41], which utilizes an invariant set normalization method where the array with median overall intensity is chosen as the baseline for normalization. Model based expressions were computed for each array and probe set using only perfect match probes.

ANOVA with p-value ≤0.01 was performed to compare the 3 subgroups based on *IKZF1* genomic status as evaluated by SNP: *IKZF1*-deletion positive (i.e. cases displaying any focal deletion in *IKZF1)*, *IKZF1*-negative cases (cases without *IKZF1* deletions), cases with chromosome 7 monosomy. A t-test with p≤0.05 and fold change difference ≥1.5 was then used to compare only *IKZF1*-deletion positive with *IKZF1*-deletion negative cases. False discovery rate (FDR) was performed over 250 permutations. A functional annotation analysis was applied to the list of selected genes using the DAVID software (http://david.abcc.ncifcrf.gov) [42] based on Gene Ontology terms. Moreover, a functional enrichment analysis was performed using the Metacore software (GeneGo Inc., www.genego.com) [43].

### Western Blot Analysis

Cells were lysed in RIPA buffer supplemented with phosphatase inhibitors. Western blots were performed according to Invitrogen procedures for NuPAGE Novex Bis-Tris Gel Electrophoresis system (Invitrogen), using 100 µg of whole-cell extracts. The antibodies used were: pStat5 and γ-H2AX antibodies (Cell Signaling Technology); Stat5 (Millipore); β-ACTIN (Sigma-Aldrich).

### Quantitative PCR analysis for *Bcl-xl*

The expression of *Bcl-xl* was examined using the following primers: F1∶5′-TGCGTGGAAAGCGTAGACAAGG-3′ and R1∶5′-CAAGGCTCTAGGTGGTCATTCAGG-3′ and a Sybr Green PCR master Mix (Applied Biosystems, Foster City, CA) on a 7900 Real-Time PCR system. The quantitative PCR thermal protocol consisted of: 50°C for 2 minutes, followed by 95°C for 10 minutes, then 40 cycles of 95°C for 1 minute and 60°C for 1 minute. *GAPDH* was used as control gene and the Stratagene's Universal Human Reference RNA was used as calibrator (Stratagene). The expression of *Bcl-xl* was assessed in 12 B-ALL patients with Ikaros deletion and in 10 B-ALL patients with wild-type Ikaros.

### *JAK2* Mutation Screening

*JAK2* mutation screening was performed on cDNA samples from 33 patients (17 *BCR-ABL1* positive and 16 *BCR-ABL1* negative cases) using the following pair primers: F1∶5′- AACTGTCATGGCCCAATTTC-3′ and R1∶5′- TGGCACATACATTCCCATGA-3′ and F2∶5′- TTGCTAAACAGTTGGCATGG-3′ and R2: CACATCTCCACACTCCCAAA-3′. cDNA was amplified with 2U of FastStart Taq DNA Polymerase (Roche Diagnostics), 0.8 mM dNTPs, 1 mM MgCl2, and 0.2 M forward and reverse primers in 25 µl reaction volumes. PCR products were purified using QIAquick PCR purification kit (Qiagen) and then directly sequenced using an ABI PRISM 3730 automated DNA sequencer (Applied Biosystems) and a Big Dye Terminator DNA sequencing kit (Applied Biosystems). All sequence variations were detected by comparison using the BLAST software tool (www.ncbi.nlm.nih.gov/BLAST/) to reference genome sequence data obtained from the UCSC browser (http://genome.ucsc.edu/cgi-bin/hgBlat?command=start; February 2009 release).

### *In vivo* Chromatin Immunoprecipitation (ChIP) Assay

2×10^7^ SD1 and BV173 cell lines and 2×10^7^ primary cultured cells from two patients with ALL were used for each immunoprecipitation. Genomic DNA was sonicated to obtain fragments in the range of 200–500 bp. The lysate was pre-cleared by incubating it with 50 µl of Immobilized Protein A (Pierce) for 15 minutes in the cold room at constant rotation. 5 µg of specific antibody were added and incubated O/N in the cold room at constant rotation. DNA fragments were amplified by Real-time quantitative PCR (RQ-PCR) using the iQ™ SYBR Green Supermix and the iQCycler thermocycler (Bio-Rad). 1/50 of the resuspended IP-DNA, was used in each PCR reaction. Antibodies employed in this study were: IgG sc-2027 and Ikaros sc-13039 (Santa Cruz Biotechnology). Specific pairs of primers used for quantitative ChIP are listed in **Table S7**.

### Luciferase Assay

The *EBF1*, *MSH2* and *MCL1* promoters were amplified by PCR and cloned into the pGL3-basic vector (Promega). The Renilla–TK vector was used as an internal control. HEK-293 cells were transiently transfected using Lipofectamine2000 (Invitrogen) according to manufacturer’s instructions. The luciferase activity was analyzed 48 h after transfection according to Dual Luciferase Assay manufacturer’s instructions (Promega).

## Supporting Information

Figure S1 **Biological process networks overrepresented in the list of genes down-regulated (A) and up-regulated (B) in B-ALL patients with** ***IKZF1*** **deletion compared to wild-type patients.** Enrichment analysis was performed using metacore-mapping software (GeneGo Inc.). Log of p-values represent the probability of a given number of genes being associated with each network by chance.
(TIF)

Figure S2 **Ikaros does not associate with promoters of the** ***MSH6, BTK, VPREB1*** **and** ***CD22*** **genes.** ChIP assay was performed in SD-1 and in BV-173 cell lines, expressing full-length Ikaros transcription factor and deleted isoform IK6, respectively. Results represent the average of three independent experiments in which each region was amplified by qPCR in triplicate. Standard error is indicated. Promoter diagram: bent arrow, transcription start site; black arrow, Ikaros binding sites; open boxes, amplicons indicated with a capital letter; chromosome and coordinates (bp) are also given.
(TIF)

Figure S3 **A large group of target promoters are not bound in vivo by deleted Ikaros in cells from a B-ALL patient.** ChIP assay was performed as described in SD-1 and in BV-173 cell lines.
(TIF)

Table S1 **Characteristics of the patients analyzed by gene expression profiling.**
(DOCX)

Table S2 **List of the down-regulated genes in** ***IKZF1*** **-deleted B-ALL patients (p<0.05).**
(DOC)

Table S3 **List of the up-regulated genes in** ***IKZF1*** **-deleted B-ALL patients (p<0.05).**
(DOC)

Table S4 **Treatment outcome and results of therapy related to** ***IKZF1*** **loss in univariate analysis.** Abbreviations: mths (months), wt (wild-type).
(DOCX)

Table S5 ***IKZF1*** **deletion and other clinical relevant factors for predicting Disease Free Survival (multivariate analysis).** Abbreviations: WBC (white blood cells).
(DOCX)

Table S6 **Description of GIMEMA clinical trials enrolling patients who were analyzed in this study.**
(DOC)

Table S7 **Specific pairs of primers used for quantitative ChIP analysis.** Different amplicons were analyzed for each gene. Abbreviations: F (forward), R (reverse).
(DOCX)

Appendix S1 **Authors of GIMEMA contributing to the study.**
(DOCX)
