# Supplementary figures and images for: IKAROS Deletions Dictate a Unique Gene Expression Signature in Patients with Adult B-Cell Acute Lymphoblastic Leukemia
Source: PLoS One. 2012 Jul 25;7(7):e40934. doi: 10.1371/journal.pone.0040934 (PMC3405023; doi:10.1371/journal.pone.0040934)

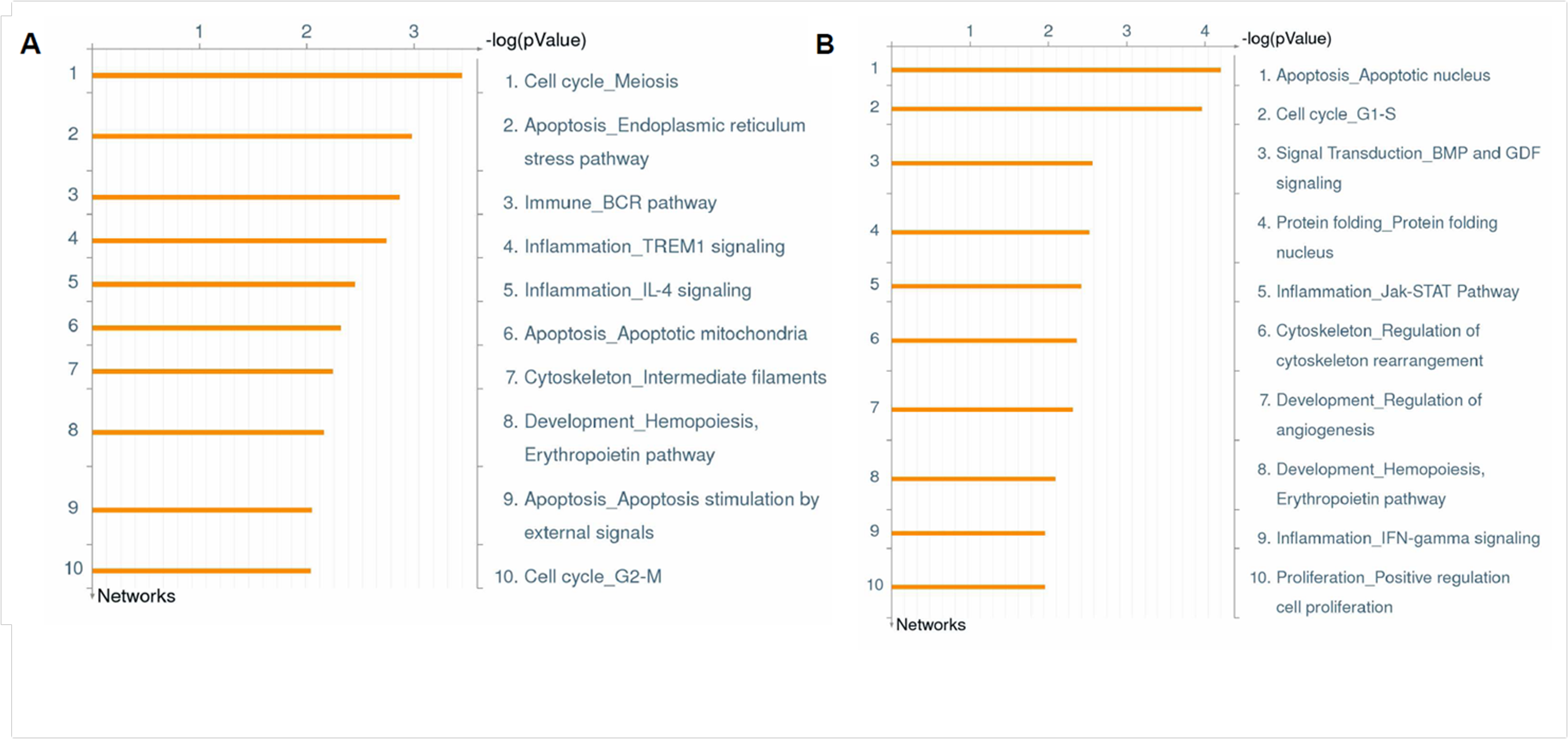

Supplement: Figure S1 — Biological process networks overrepresented in the list of genes down-regulated (A) and up-regulated (B) in B-ALL patients with IKZF1 deletion compared to wild-type patients. Enrichment analysis was performed using metacore-mapping software (GeneGo Inc.). Log of p-values represent the probability of a given number of genes being associated with each network by chance. (TIF) [file pone.0040934.s001.tif]

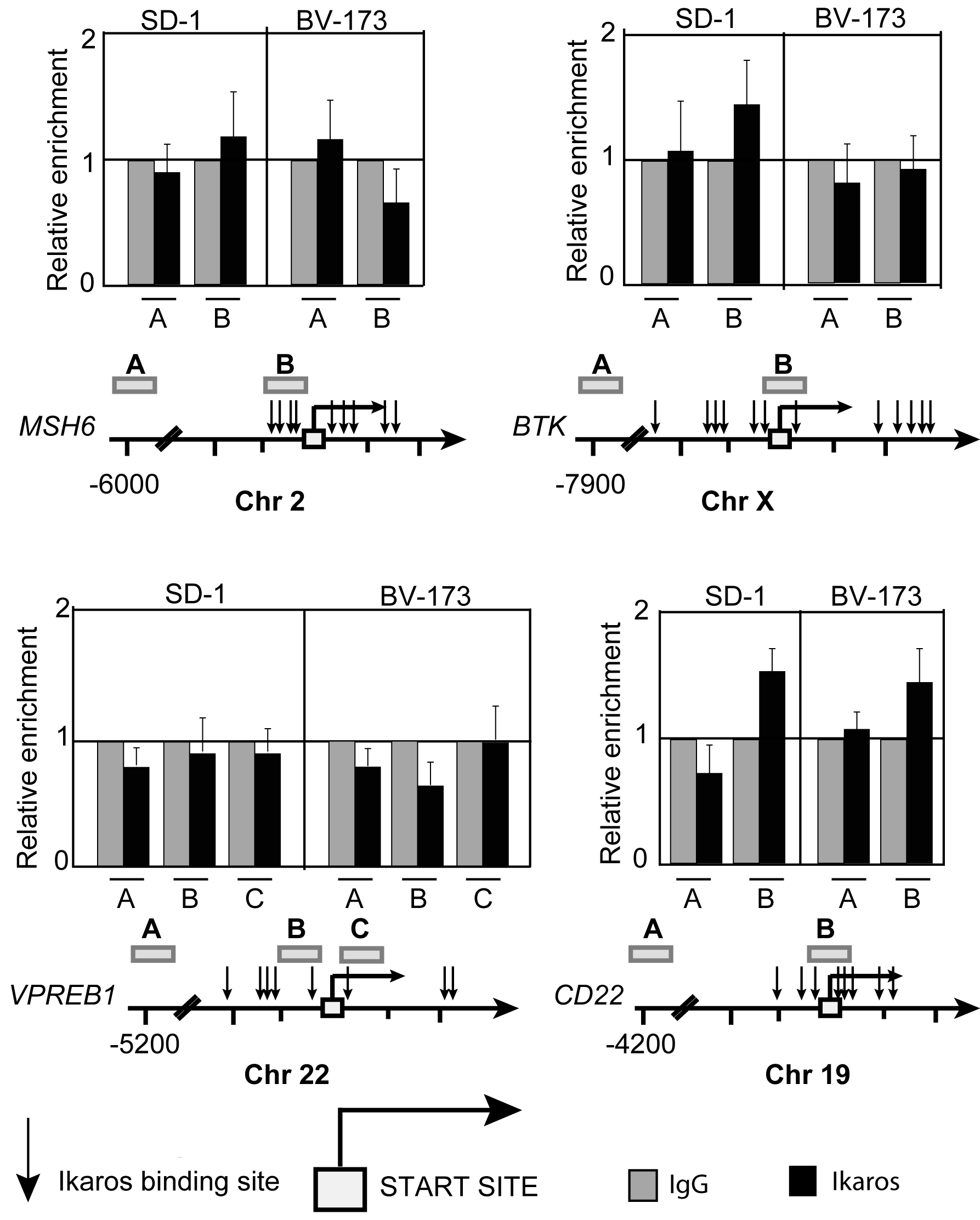

Supplement: Figure S2 — Ikaros does not associate with promoters of the MSH6, BTK, VPREB1 and CD22 genes. ChIP assay was performed in SD-1 and in BV-173 cell lines, expressing full-length Ikaros transcription factor and deleted isoform IK6, respectively. Results represent the average of three independent experiments in which each region was amplified by qPCR in triplicate. Standard error is indicated. Promoter diagram: bent arrow, transcription start site; black arrow, Ikaros binding sites; open boxes, amplicons indicated with a capital letter; chromosome and coordinates (bp) are also given. (TIF) [file pone.0040934.s002.tif]

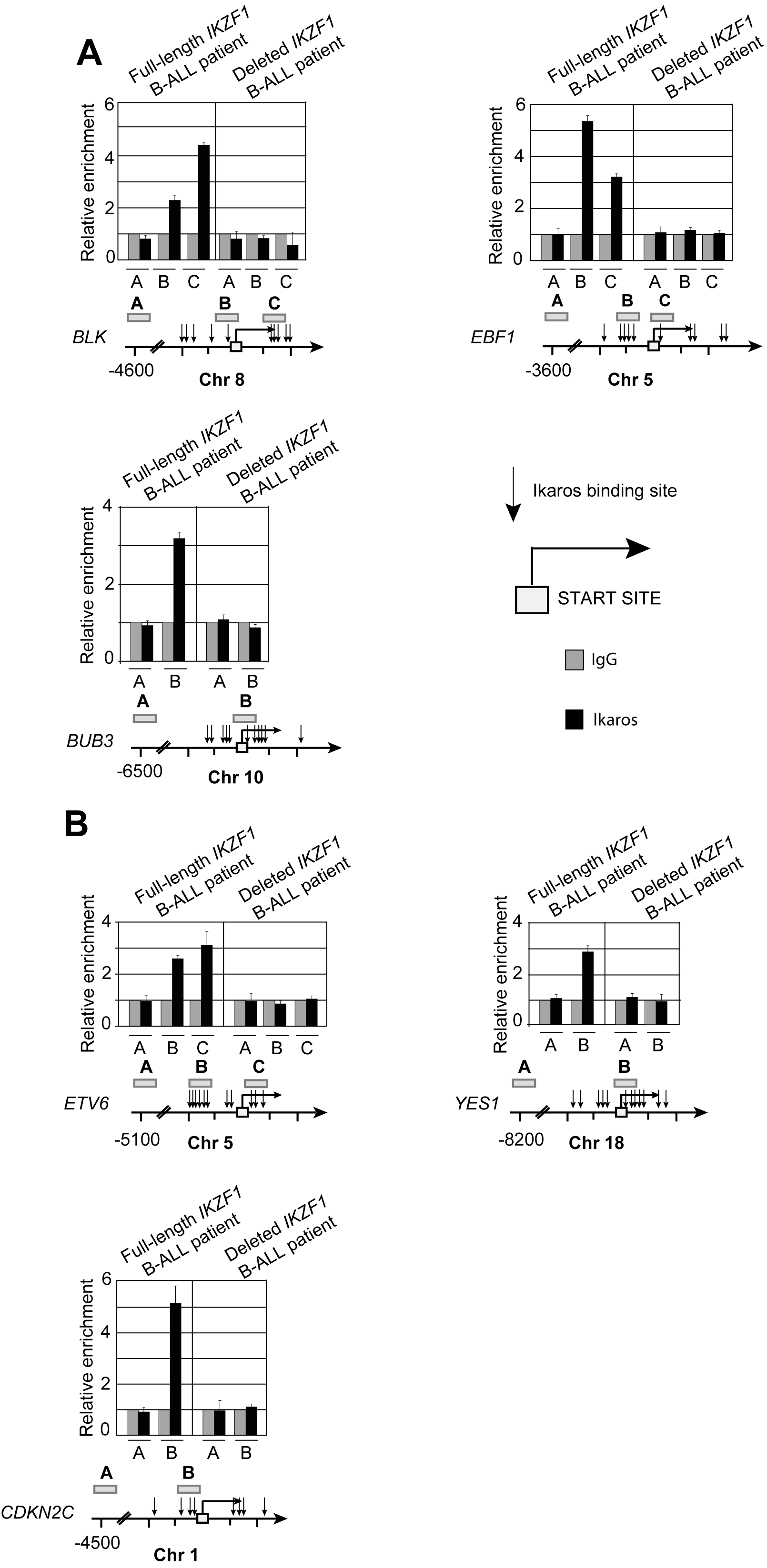

Supplement: Figure S3 — A large group of target promoters are not bound in vivo by deleted Ikaros in cells from a B-ALL patient. ChIP assay was performed as described in SD-1 and in BV-173 cell lines. (TIF) [file pone.0040934.s003.tif]
