# Supplementary material for: IKAROS Deletions Dictate a Unique Gene Expression Signature in Patients with Adult B-Cell Acute Lymphoblastic Leukemia
Source: PLoS One. 2012 Jul 25;7(7):e40934. doi: 10.1371/journal.pone.0040934 (PMC3405023; doi:10.1371/journal.pone.0040934)
Supplement: Table S1 — Characteristics of the patients analyzed by gene expression profiling. (DOCX) [file pone.0040934.s004.docx]

| **Patient Characteristics** | ***BCR-ABL1*+ ALL** | **B-NEG ALL** |  |
| --- | --- | --- | --- |
| Number | 30 | 32 |  |
| Age, years  Median  Range | 53  (18 - 76) | 31  (16-53) |  |
| Blast, %  Median  Range | 90  (18-99) | 94.5  (80-100) |  |
| Sex  Male (%)  Female (%) | 15 (50)  15 (50) | 23 (72)  9 (28) |  |
| *IKZF1* deletion | 16 (53) | 15 (47) |  |
| Chr 7 Monosomy | 8 (25) | 3 (9) |  |
