# Supplementary material for: IKAROS Deletions Dictate a Unique Gene Expression Signature in Patients with Adult B-Cell Acute Lymphoblastic Leukemia
Source: PLoS One. 2012 Jul 25;7(7):e40934. doi: 10.1371/journal.pone.0040934 (PMC3405023; doi:10.1371/journal.pone.0040934)
Supplement: Table S2 — List of the down-regulated genes in IKZF1 -deleted B-ALL patients (p<0.05). (DOC) [file pone.0040934.s005.doc]

| **Probe set** | **Gene** | **Accession** | **P value** | **LocusLink** | **Gene Symbol** | **Cytoband** |
| --- | --- | --- | --- | --- | --- | --- |
| 203556_at | zinc fingers and homeoboxes 2 | NM_014943 | 0.00003 | 22882 | ZHX2 | 8q24.13 |
| 218147_s_at | glycosyltransferase 8 domain containing 1 | NM_018446 | 0.000046 | 55830 | GLT8D1 | 3p21.1 |
| 218146_at | glycosyltransferase 8 domain containing 1 | NM_018446 | 0.000148 | 55830 | GLT8D1 | 3p21.1 |
| 204236_at | Friend leukemia virus integration 1 | NM_002017 | 0.000256 | 2313 | FLI1 | 11q24.1-q24.3 |
| 39318_at | T-cell leukemia/lymphoma 1A | X82240 | 0.000256 | 8115 | TCL1A | 14q32.1 |
| 211962_s_at | zinc finger protein 36. C3H type-like 1 | BG250310 | 0.000264 | 677 | ZFP36L1 | 14q22-q24 |
| 210786_s_at | Friend leukemia virus integration 1 | M93255 | 0.000304 | 2313 | FLI1 | 11q24.1-q24.3 |
| 209995_s_at | T-cell leukemia/lymphoma 1A | BC003574 | 0.000314 | 8115 | TCL1A | 14q32.1 |
| 218285_s_at | dehydrogenase/reductase (SDR family) member 6 | NM_020139 | 0.00034 | 56898 | BDH2 | 4q24 |
| 201054_at | heterogeneous nuclear ribonucleoprotein A0 | BE966599 | 0.000653 | 10949 | HNRNPA0 | 5q31 |
| 218973_at | elongation factor Tu GTP binding domain containing 1 | NM_024580 | 0.000688 | 79631 | EFTUD1 | 15q25.2 |
| 225136_at | Pleckstrin homology domain containing family A (phosphoinositide binding specific) member 2 | BF968578 | 0.000811 | 59339 | PLEKHA2 | 8p11.23 |
| 223273_at | chromosome 14 open reading frame 142 | AF277185 | 0.000858 | 84520 | C14orf142 | 14q32.12 |
| 201458_s_at | BUB3 budding uninhibited by benzimidazoles 3 homolog (yeast) | NM_004725 | 0.001002 | 9184 | BUB3 | 10q26 |
| 217718_s_at | tyrosine 3-monooxygenase/tryptophan 5-monooxygenase activation protein. beta polypeptide | NM_014052 | 0.001056 | 7529 | YWHAB | 20q13.1 |
| 241933_at | Glutaminyl-tRNA synthase (glutamine-hydrolyzing)-like 1 | AA769438 | 0.001574 | 55278 | QRSL1 | 6q21 |
| 218949_s_at | Glutaminyl-tRNA synthase (glutamine-hydrolyzing)-like 1 | NM_018292 | 0.001705 | 55278 | QRSL1 | 6q21 |
| 201700_at | cyclin D3 | NM_001760 | 0.001715 | 896 | CCND3 | 6p21 |
| 226184_at | formin-like 2 | AI123567 | 0.002163 | 114793 | FMNL2 | 2q23.3 |
| 218641_at | hypothetical protein MGC3032 | NM_023941 | 0.002433 | 65998 | MGC3032 | 11q13 |
| 202266_at | TRAF and TNF receptor associated protein | NM_016614 | 0.002492 | 51567 | TTRAP | 6p22.3-p22.1 |
| 226254_s_at | KIAA1430 | AI912523 | 0.002763 | 57587 | KIAA1430 | 4q35.1 |
| 218970_s_at | cutC copper transporter homolog (E.coli) | NM_015960 | 0.002807 | 51076 | CUTC | 10q24.2 |
| 219244_s_at | mitochondrial ribosomal protein L46 | NM_022163 | 0.002852 | 26589 | MRPL46 | 15q24-q25 |
| 202806_at | drebrin 1 | NM_004395 | 0.002872 | 1627 | DBN1 | 5q35.3 |
| 38521_at | CD22 antigen | X59350 | 0.003117 | 933 | CD22 | 19q13.1 |
| 204581_at | CD22 antigen | NM_001771 | 0.003139 | 933 | CD22 | 19q13.1 |
| 228752_at | chromosome X open reading frame 10 | N57931 | 0.003141 | 93949 | BCORL1 | Xq25-q26.1 |
| 45633_at | hypothetical protein FLJ13912 | AI421812 | 0.003143 | 64785 | GINS3 | 16q21 |
| 209096_at | ubiquitin-conjugating enzyme E2 variant 2 | U62136 | 0.003151 | 7336 | UBE2V2 | 8q11.21 |
| 201792_at | AE binding protein 1 | NM_001129 | 0.003194 | 165 | AEBP1 | 7p13 |
| 205504_at | Bruton agammaglobulinemia tyrosine kinase | NM_000061 | 0.003199 | 695 | BTK | Xq21.33-q22 |
| 203259_s_at | chromosome 6 open reading frame 74 | BC001671 | 0.00339 | 51020 | HDDC2 | 6q13-q24.3 |
| 211023_at | pyruvate dehydrogenase (lipoamide) beta | AL117618 | 0.003586 | 5162 | PDHB | 3p21.1-p14.2 |
| 218123_at | chromosome 21 open reading frame 59 | NM_017835 | 0.003601 | 56683 | C21orf59 | 21q22.1 |
| 220643_s_at | Fas apoptotic inhibitory molecule | NM_018147 | 0.003668 | 55179 | FAIM | 3q22.3 |
| 225019_at | calcium/calmodulin-dependent protein kinase (CaM kinase) II delta | BF797381 | 0.003723 | 817 | CAMK2D | 4q26 |
| 224437_s_at | chromosome 6 open reading frame 55 | BC005937 | 0.003833 | 51534 | VTA1 | 6q24.1 |
| 201142_at | Eukaryotic translation initiation factor 2. subunit 1 alpha. 35kDa | AA577698 | 0.003899 | 1965 | EIF2S1 | 14q23.3 |
| 203856_at | vaccinia related kinase 1 | NM_003384 | 0.003985 | 7443 | VRK1 | 14q32 |
| 227413_at | hypothetical protein MGC10067 | BF965546 | 0.004099 | 134510 | UBLCP1 | 5q33.3 |
| 225391_at | hypothetical protein BC006130 | AL562398 | 0.004133 | 93622 | LOC93622 | 4p16.1 |
| 208939_at | selenophosphate synthetase 1 | AV682679 | 0.004573 | 22929 | SEPHS1 | 10p14 |
| 208940_at | Selenophosphate synthetase 1 | AI885670 | 0.004574 | 22929 | SEPHS1 | 10p14 |
| 204185_x_at | peptidylprolyl isomerase D (cyclophilin D) | NM_005038 | 0.004579 | 5481 | PPID | 4q31.3 |
| 221969_at | Paired box gene 5 (B-cell lineage specific activator) | BF510692 | 0.004713 | 5079 | --- | --- |
| 225841_at | hypothetical protein FLJ30525 | BE502436 | 0.004715 | 113802 | C1orf59 | 1p13.3 |
| 1556599_s_at | Hypothetical LOC389105 | AI698023 | 0.004972 | 389105 | ARPP-21 /// LOC100130503 | 3p22.3 |
| 203276_at | lamin B1 | NM_005573 | 0.005276 | 4001 | LMNB1 | 5q23.3-q31.1 |
| 221234_s_at | BTB and CNC homology 1. basic leucine zipper transcription factor 2 | NM_021813 | 0.005432 | 60468 | BACH2 | 6q15 |
| 226796_at | hypothetical protein LOC116236 | AI817418 | 0.00547 | 116236 | LOC116236 | 17q11.2 |
| 225633_at | hypothetical protein LOC147991 | BF057717 | 0.006069 | 147991 | DPY19L3 | 19q13.11 |
| 223337_at | serologically defined colon cancer antigen 10 | AF039693 | 0.00621 | 10283 | SDCCAG10 | 5q12.3 |
| 208696_at | chaperonin containing TCP1. subunit 5 (epsilon) | AF275798 | 0.006345 | 22948 | CCT5 | 5p15.2 |
| 208639_x_at | protein disulfide isomerase-associated 6 | BC001312 | 0.006559 | 10130 | PDIA6 | 2p25.1 |
| 201877_s_at | protein phosphatase 2 regulatory subunit B (B56). gamma isoform | NM_002719 | 0.00665 | 5527 | PPP2R5C | 14q32 |
| 218592_s_at | cat eye syndrome chromosome region. candidate 5 | NM_017829 | 0.006747 | 27440 | CECR5 | --- |
| 210450_at | hypothetical protein LOC90925 | BC002792 | 0.007015 | 90925 | LOC90925 | 14q32.33 |
| 218719_s_at | hypothetical protein FLJ13912 | NM_022770 | 0.007037 | 64785 | GINS3 | 16q21 |
| 204735_at | phosphodiesterase 4A. cAMP-specific (phosphodiesterase E2 dunce homolog. Drosophila) | NM_006202 | 0.007049 | 5141 | PDE4A | 19p13.2 |
| 200848_at | S-adenosylhomocysteine hydrolase-like 1 | AA479488 | 0.007513 | 10768 | AHCYL1 | 1p13.2 |
| 211725_s_at | BH3 interacting domain death agonist | BC005884 | 0.007907 | 637 | BID | 22q11.1 |
| 212199_at | Morf4 family associated protein 1-like 1 | AL566962 | 0.008012 | 114932 | MRFAP1L1 | 4p16.1 |
| 1552691_at | ADP-ribosylation factor-like 11 | NM_138450 | 0.008488 | 115761 | ARL11 | 13q14.3 |
| 225086_at | Hypothetical protein FLJ38426 | BF679966 | 0.008644 | 283742 | FAM98B | 15q14 |
| 228333_at | Full length insert cDNA clone YT94E02 | AI912571 | 0.008872 |  | --- | --- |
| 221589_s_at | Aldehyde dehydrogenase 6 family. member A1 | AW612403 | 0.008939 | 4329 | ALDH6A1 | 14q24.3 |
| 211965_at | zinc finger protein 36 C3H type-like 1 | BE620915 | 0.009158 | 677 | ZFP36L1 | 14q22-q24 |
| 204897_at | prostaglandin E receptor 4 (subtype EP4) | AA897516 | 0.010134 | 5734 | PTGER4 | 5p13.1 |
| 222029_x_at | HLA class II region expressed gene KE2 | NM_014260 | 0.010285 | 10471 | PFDN6 | 6p21.3 |
| 215946_x_at | PREDICTED: Homo sapiens similar to omega protein (LOC91353) mRNA | AL022324 | 0.010488 |  | IGLL3 | 22q11.2|22q11.23 |
| 218826_at | solute carrier family 35. member F2 | NM_017515 | 0.010831 | 54733 | SLC35F2 | 11q22.3 |
| 207677_s_at | neutrophil cytosolic factor 4 40kDa | NM_013416 | 0.010863 | 4689 | NCF4 | 22q13.1 |
| 203603_s_at | zinc finger homeobox 1b | NM_014795 | 0.011263 | 9839 | ZEB2 | 2q22 |
| 224392_s_at | gb:AF303588.1 /DB_XREF=gi:13649589 /GEN=OPN3 /FEA=FLmRNA /CNT=1 /TID=HsAffx.900585.620 /TIER=FL /STK=0 /DEF=Homo sapiens panopsin (OPN3) mRNA. complete cds. /PROD=panopsin /FL=gb:AF303588.1 | AF303588 | 0.011278 |  | OPN3 | 1q43 |
| 223088_x_at | enoyl Coenzyme A hydratase domain containing 1 | BC003549 | 0.011563 | 55862 | ECHDC1 | 6q22.33 |
| 227173_s_at | BTB and CNC homology 1 basic leucine zipper transcription factor 2 | AW450901 | 0.012053 | 60468 | BACH2 | 6q15 |
| 226666_at | Dishevelled associated activator of morphogenesis 1 | BG434703 | 0.012117 | 23002 | DAAM1 | 14q23.1 |
| 219974_x_at | enoyl Coenzyme A hydratase domain containing 1 | NM_018479 | 0.01217 | 55862 | ECHDC1 | 6q22.33 |
| 242521_at | CDNA clone IMAGE:30349460. partial cds | BF512556 | 0.012982 |  | --- | --- |
| 233588_x_at | HLA class II region expressed gene KE2 | BE561798 | 0.013148 | 10471 | PFDN6 | 6p21.3 |
| 239214_at | Hypothetical gene supported by AK091718 | AA806831 | 0.013186 | 401504 | LOC100130458 | 9p13.2 |
| 204613_at | phospholipase C. gamma 2 (phosphatidylinositol-specific) | NM_002661 | 0.013356 | 5336 | PLCG2 | 16q24.1 |
| 204205_at | apolipoprotein B mRNA editing enzyme. catalytic polypeptide-like 3G | NM_021822 | 0.013399 | 60489 | APOBEC3G | 22q13.1-q13.2 |
| 209421_at | mutS homolog 2 colon cancer nonpolyposis type 1 (E. coli) | U04045 | 0.013481 | 4436 | MSH2 | 2p22-p21 |
| 244261_at | interleukin 28 receptor alpha (interferon. lambda receptor) | AW340139 | 0.014247 | 163702 | IL28RA | 1p36.11 |
| 222416_at | aldehyde dehydrogenase 18 family member A1 | U76542 | 0.014289 | 5832 | ALDH18A1 | 10q24.3 |
| 201143_s_at | eukaryotic translation initiation factor 2 subunit 1 alpha 35kDa | BC002513 | 0.014511 | 1965 | EIF2S1 | 14q23.3 |
| 204905_s_at | eukaryotic translation elongation factor 1 epsilon 1 | NM_004280 | 0.01498 | 9521 | EEF1E1 | 6p24.3-p25.1 |
| 205147_x_at | neutrophil cytosolic factor 4. 40kDa | NM_000631 | 0.015301 | 4689 | NCF4 | 22q13.1 |
| 202911_at | mutS homolog 6 (E. coli) | NM_000179 | 0.015306 | 2956 | MSH6 | 2p16 |
| 219032_x_at | opsin 3 (encephalopsin. panopsin) | NM_014322 | 0.015534 | 23596 | OPN3 | 1q43 |
| 216060_s_at | dishevelled associated activator of morphogenesis 1 | AK021890 | 0.015853 | 23002 | DAAM1 | 14q23.1 |
| 209900_s_at | solute carrier family 16 (monocarboxylic acid transporters) member 1 | AL162079 | 0.016335 | 6566 | SLC16A1 | 1p12 |
| 224994_at | calcium/calmodulin-dependent protein kinase (CaM kinase) II delta | AA777512 | 0.016358 | 817 | CAMK2D | 4q26 |
| 228594_at | hypothetical protein FLJ30596 | H94910 | 0.016536 | 133686 | C5orf33 | 5p13.2 |
| 200703_at | dynein cytoplasmic light polypeptide 1 | NM_003746 | 0.01728 | 8655 | DYNLL1 | 12q24.23 |
| 229670_at | 5.5 kb mRNA upregulated in retinoic acid treated HL-60 neutrophilic cells | BF056369 | 0.017393 |  | --- | --- |
| 238021_s_at | gb:AA954994 /DB_XREF=gi:3118689 /DB_XREF=op24f03.s1 /CLONE=IMAGE:1577789 /FEA=EST /CNT=15 /TID=Hs.237396.1 /TIER=ConsEnd /STK=1 /UG=Hs.237396 /UG_TITLE=ESTs | AA954994 | 0.017795 |  | hCG_1815491 | 16q12.2 |
| 224880_at | v-ral simian leukemia viral oncogene homolog A (ras related) | AV703462 | 0.017989 | 5898 | RALA | 7p15-p13 |
| 238022_at | Hypothetical gene supported by AF275804 | AA954994 | 0.018327 | 388279 | hCG_1815491 | 16q12.2 |
| 225927_at | mitogen-activated protein kinase kinase kinase 1 | AA541479 | 0.019096 | 4214 | MAP3K1 | 5q11.2 |
| 205449_at | SAC3 domain containing 1 | NM_013299 | 0.019653 | 29901 | SAC3D1 | 11q13.1 |
| 213358_at | KIAA0802 | AB018345 | 0.020578 | 23255 | KIAA0802 | 18p11.22 |
| 233827_s_at | suppressor of Ty 16 homolog (S. cerevisiae) | AK024072 | 0.020876 | 11198 | SUPT16H | 14q11.2 |
| 203335_at | phytanoyl-CoA hydroxylase (Refsum disease) | NM_006214 | 0.021357 | 5264 | PHYH | 10p13 |
| 218049_s_at | mitochondrial ribosomal protein L13 | NM_014078 | 0.022042 | 28998 | MRPL13 | 8q22.1-q22.3 |
| 222388_s_at | vacuolar protein sorting 35 (yeast) | AF186382 | 0.022492 | 55737 | VPS35 | 16q12 |
| 201129_at | splicing factor arginine/serine-rich 7 35kDa | NM_006276 | 0.022564 | 6432 | SFRS7 | 2p22.1 |
| 218935_at | EH-domain containing 3 | NM_014600 | 0.02284 | 30845 | EHD3 | 2p21 |
| 228597_at | chromosome 21 open reading frame 45 | AW151538 | 0.023595 | 54069 | C21orf45 | 21q22.11 |
| 203386_at | TBC1 domain family member 4 | AI650848 | 0.024368 | 9882 | TBC1D4 | 13q22.2 |
| 212604_at | mitochondrial ribosomal protein S31 | AI937794 | 0.025025 | 10240 | MRPS31 | 13q14.11 |
| 228555_at | Calcium/calmodulin-dependent protein kinase (CaM kinase) II delta | AA029441 | 0.025576 | 817 | CAMK2D | 4q26 |
| 227198_at | Lymphoid nuclear protein related to AF4 | AW085505 | 0.025984 | 3899 | AFF3 | 2q11.2-q12 |
| 242665_at | formin-like 2 | AL042120 | 0.026087 | 114793 | FMNL2 | 2q23.3 |
| 201310_s_at | chromosome 5 open reading frame 13 | NM_004772 | 0.02722 | 9315 | C5orf13 | 5q22.1 |
| 202532_s_at | dihydrofolate reductase | BC000192 | 0.027618 | 1719 | DHFR | 5q11.2-q13.2 |
| 224576_at | endoplasmic reticulum-golgi intermediate compartment 32 kDa protein | AK000752 | 0.028404 | 57222 | ERGIC1 | 5q35.1-q35.2 |
| 218573_at | melanoma antigen family H. 1 | NM_014061 | 0.028567 | 28986 | MAGEH1 | Xp11.21 |
| 203685_at | B-cell CLL/lymphoma 2 | NM_000633 | 0.028621 | 596 | BCL2 | 18q21.33|18q21.3 |
| 238919_at | Protocadherin 9 | R49295 | 0.029378 | 5101 | --- | --- |
| 225367_at | phosphoglucomutase 2 | BF512139 | 0.030098 | 55276 | PGM2 | 4p14 |
| 216080_s_at | fatty acid desaturase 3 | AC004770 | 0.030149 | 3995 | FADS3 | 11q12-q13.1 |
| 207920_x_at | zinc finger protein X-linked | NM_003410 | 0.030353 | 7543 | ZFX | Xp21.3 |
| 205590_at | RAS guanyl releasing protein 1 (calcium and DAG-regulated) | NM_005739 | 0.031263 | 10125 | RASGRP1 | 15q14 |
| 200644_at | MARCKS-like 1 | NM_023009 | 0.031444 | 65108 | MARCKSL1 | 1p35.1 |
| 213129_s_at | glycine cleavage system protein H (aminomethyl carrier) | AI970157 | 0.031881 | 2653 | GCSH /// LOC730107 | 16q23.2 /// 1q24.2 |
| 213133_s_at | glycine cleavage system protein H (aminomethyl carrier) | AW237404 | 0.032171 | 2653 | GCSH /// LOC730107 | 16q23.2 /// 1q24.2 |
| 209670_at | T cell receptor alpha constant | M12959 | 0.032199 | 28755 | TRAC | 14q11 |
| 200614_at | clathrin heavy polypeptide (Hc) | NM_004859 | 0.032307 | 1213 | CLTC | 17q11-qter |
| 202733_at | procollagen-proline 2-oxoglutarate 4-dioxygenase (proline 4-hydroxylase) alpha polypeptide II | NM_004199 | 0.032359 | 8974 | P4HA2 | 5q31 |
| 1565602_at | Protocadherin 9 | AF085861 | 0.03255 | 5101 | --- | --- |
| 211450_s_at | mutS homolog 6 (E. coli) | D89646 | 0.033019 | 2956 | MSH6 | 2p16 |
| 223060_at | chromosome 14 open reading frame 119 | AF061731 | 0.033199 | 55017 | C14orf119 | 14q11.2 |
| 217853_at | tensin-like SH2 domain containing 1 | NM_022748 | 0.03513 | 64759 | TNS3 | 7p12.3 |
| 224762_at | tumor differentially expressed 2-like | AA872583 | 0.035745 | 347735 | KIAA0746 /// SERINC2 | 1p35.1 /// 4p15.2 |
| 214435_x_at | v-ral simian leukemia viral oncogene homolog A (ras related) | NM_005402 | 0.035992 | 5898 | RALA | 7p15-p13 |
| 201563_at | sorbitol dehydrogenase | L29008 | 0.036002 | 6652 | SORD | 15q15.3 |
| 235085_at | hypothetical protein DKFZp761P0423 | BF739767 | 0.036053 | 157285 | PRAGMIN | 8p23.1 |
| 1552767_a_at | heparan sulfate 6-O-sulfotransferase 2 | NM_147174 | 0.036784 | 90161 | HS6ST2 | Xq26.2 |
| 206660_at | immunoglobulin lambda-like polypeptide 1 | NM_020070 | 0.036802 | 3543 | IGLL1 | 22q11.23 |
| 202345_s_at | fatty acid binding protein 5 (psoriasis-associated) | NM_001444 | 0.03696 | 2171 | FABP5 /// FABP5L2 /// FABP5L7 | 11q12.1 /// 13q14.3 /// 8q21.13 |
| 201072_s_at | SWI/SNF related. matrix associated. actin dependent regulator of chromatin subfamily cmember 1 | AW152160 | 0.037218 | 6599 | SMARCC1 | 3p23-p21 |
| 219218_at | KIAA1447 protein | NM_024696 | 0.037677 | 57597 | BAHCC1 | 17q25.3 |
| 203744_at | high-mobility group box 3 | NM_005342 | 0.038197 | 3149 | HMGB3 | Xq28 |
| 1555501_s_at | arginine/serine-rich coiled-coil 1 | BC010357 | 0.038232 | 51319 | RSRC1 | 3q25.32 |
| 210944_s_at | calpain 3. (p94) | BC003169 | 0.038858 | 825 | CAPN3 | 15q15.1-q21.1 |
| 206255_at | B lymphoid tyrosine kinase | NM_001715 | 0.039904 | 640 | BLK | 8p23-p22 |
| 209715_at | chromobox homolog 5 (HP1 alpha homolog. Drosophila) | L07515 | 0.040748 | 23468 | CBX5 | 12q13.13 |
| 230030_at | heparan sulfate 6-O-sulfotransferase 2 | AI767756 | 0.040968 | 90161 | HS6ST2 | Xq26.2 |
| 221021_s_at | catenin. beta like 1 | NM_030877 | 0.041015 | 56259 | CTNNBL1 | 20q11.23-q12 |
| 224428_s_at | cell division cycle associated 7 | AY029179 | 0.0413 | 83879 | CDCA7 | 2q31 |
| 201761_at | methylenetetrahydrofolate dehydrogenase (NADP+ dependent) 2 methenyltetrahydrofolate cyclohydrolase | NM_006636 | 0.042874 | 10797 | MTHFD2 | 2p13.1 |
| 226436_at | Ras association (RalGDS/AF-6) domain family 4 | N49935 | 0.044355 | 83937 | RASSF4 | 10q11.21 |
| 213502_x_at | similar to bK246H3.1 (immunoglobulin lambda-like polypeptide 1 pre-B-cell specific) | AA398569 | 0.04443 | 91316 | LOC91316 | 22q11.23 |
| 209123_at | quinoid dihydropteridine reductase | BC000576 | 0.045092 | 5860 | QDPR | 4p15.31 |
| 242468_at | gb:AA767317 /DB_XREF=gi:2818332 /DB_XREF=nz65f02.s1 /CLONE=IMAGE:1300347 /FEA=EST /CNT=4 /TID=Hs.291891.0 /TIER=ConsEnd /STK=3 /UG=Hs.291891 /UG_TITLE=ESTs | AA767317 | 0.047652 |  | --- | --- |
