# Supplementary material for: IKAROS Deletions Dictate a Unique Gene Expression Signature in Patients with Adult B-Cell Acute Lymphoblastic Leukemia
Source: PLoS One. 2012 Jul 25;7(7):e40934. doi: 10.1371/journal.pone.0040934 (PMC3405023; doi:10.1371/journal.pone.0040934)
Supplement: Table S3 — List of the up-regulated genes in IKZF1 -deleted B-ALL patients (p<0.05). (DOC) [file pone.0040934.s006.doc]

| **Probe set** | **Gene** | **Accession** | **P value** | **LocusLink** | **Gene Symbol** | **Cytoband** |
| --- | --- | --- | --- | --- | --- | --- |
| 221832_s_at | leucine zipper protein 1 | AV741657 | 0.000055 | 7798 | LUZP1 | 1p36 |
| 202947_s_at | glycophorin C (Gerbich blood group) | NM_002101 | 0.000067 | 2995 | GYPC | 2q14-q21 |
| 231431_s_at | Hypothetical LOC388114 | AI125670 | 0.000072 | --- | --- | --- |
| 218899_s_at | brain and acute leukemia cytoplasmic | NM_024812 | 0.000088 | 79870 | BAALC | 8q22.3 |
| 208116_s_at | mannosidase. alpha. class 1A. member 1 | NM_005907 | 0.000097 | 4121 | MAN1A1 | 6q22 |
| 226130_at | ribosomal protein S16 | AA583817 | 0.000114 | 441876 /// 6217 | LOC441876 /// RPS16 | 19q13.1 /// 1p36.21 |
| 224831_at | cytoplasmic polyadenylation element binding protein 4 | BE620832 | 0.000127 | 80315 | CPEB4 | 5q21 |
| 208724_s_at | RAB1A. member RAS oncogene family | BC000905 | 0.00015 | 5861 | RAB1A | 2p14 |
| 211474_s_at | serine (or cysteine) proteinase inhibitor clade B (ovalbumin). member 6 | BC004948 | 0.000196 | 5269 | SERPINB6 | 6p25 |
| 240671_at | Glycophorin C (Gerbich blood group) | H38635 | 0.000207 | --- | --- | --- |
| 225567_at | Hypothetical LOC388114 | BE207755 | 0.00021 | --- | --- | --- |
| 1558173_a_at | leucine zipper protein 1 | AK093016 | 0.000215 | 7798 | LUZP1 | 1p36 |
| 203839_s_at | tyrosine kinase non-receptor 2 | NM_005781 | 0.00022 | 10188 | TNK2 | 3q29 |
| 200984_s_at | CD59 antigen p18-20 (antigen identified by monoclonal antibodies 16.3A5. EJ16. EJ30. EL32 and G344) | X16447 | 0.000225 | 966 | CD59 | 11p13 |
| 213737_x_at | hypothetical LOC283768 /// golgi autoantigen. golgin family member /// similar to hypothetical protein | AI620911 | 0.000229 | 283796 | GOLGA9P | 15q11.2 |
| 225799_at | hypothetical protein MGC4677 /// hypothetical LOC541471 protein | BF209337 | 0.00023 | 112597 /// 541471 | LOC541471 /// NCRNA00152 | 2p11.2 /// 2q13 |
| 218764_at | protein kinase C eta | NM_024064 | 0.000242 | 5583 | PRKCH | 14q22-q23 |
| 202615_at | Guanine nucleotide binding protein (G protein). q polypeptide | BF222895 | 0.000261 | 2776 | GNAQ | 9q21 |
| 217783_s_at | yippee-like 5 (Drosophila) | NM_016061 | 0.000269 | 51646 | YPEL5 | 2p23.1 |
| 237849_at | Mannosidase alpha class 1A member 1 | BE674460 | 0.000307 | --- | --- | --- |
| 200985_s_at | CD59 antigen p18-20 (antigen identified by monoclonal antibodies 16.3A5. EJ16. EJ30. EL32 and G344) | NM_000611 | 0.000327 | 966 | CD59 | 11p13 |
| 1565913_at | Zinc finger CCCH type antiviral 1 | H59257 | 0.000375 | --- | --- | --- |
| 220104_at | Zinc finger CCCH type antiviral 1 | NM_020119 | 0.000387 | 56829 | ZC3HAV1 | 7q34 |
| 202393_s_at | Kruppel-like factor 10 | NM_005655 | 0.00046 | 7071 | KLF10 | 8q22.2 |
| 213075_at | olfactomedin-like 2A | AL050002 | 0.000461 | 169611 | OLFML2A | 9q33.3 |
| 238689_at | G protein-coupled receptor 110 | BG426455 | 0.000467 | 266977 | GPR110 | 6p12.3 |
| 226155_at | KIAA1600 | AB046820 | 0.000481 | 57700 | FAM160B1 | 10q25.3 |
| 226641_at | Hypothetical protein DKFZp434D2328 | AU157224 | 0.000485 | --- | --- | --- |
| 226018_at | hypothetical protein Ells1 | W73230 | 0.000508 | 222166 | C7orf41 | 7p15.1 |
| 222780_s_at | brain and acute leukemia cytoplasmic | AI870583 | 0.00053 | 79870 | BAALC | 8q22.3 |
| 226914_at | actin related protein 2/3 complex subunit 5-like | AU158936 | 0.00054 | 81873 | ARPC5L | 9q33.3 |
| 1566079_at | ELOVL family member 5. elongation of long chain fatty acids (FEN1/Elo2. SUR4/Elo3-like. yeast) | AL833001 | 0.000574 | 647190 | LOC647190 | 6p12.1 |
| 235421_at | Mitogen-activated protein kinase kinase kinase 8 | AV713062 | 0.0006 | 1326 | MAP3K8 | 10p11.23 |
| 212665_at | TCDD-inducible poly(ADP-ribose) polymerase | AL556438 | 0.000604 | 25976 | TIPARP | 3q25.31 |
| 205027_s_at | mitogen-activated protein kinase kinase kinase 8 | NM_005204 | 0.000605 | 1326 | MAP3K8 | 10p11.23 |
| 215375_x_at | CDNA FLJ13876 fis clone THYRO1001401 | AK023938 | 0.000638 | 9208 | LRRFIP1 | 2q37.3 |
| 243527_at | Mariner-like element-containing mRNA. clone pcHMT2 | AW793677 | 0.000638 | --- | --- | --- |
| 1556209_at | C-type lectin domain family 2 member B | CA447397 | 0.000659 | 9976 | CLEC2B | 12p13-p12 |
| 228456_s_at | hypothetical protein LOC149832 | AU151357 | 0.000726 | 149832 | LOC149832 | 20p13 |
| 236488_s_at | CDNA FLJ36309 fis clone THYMU2004986 | AI683805 | 0.00076 | --- | --- | --- |
| 200797_s_at | myeloid cell leukemia sequence 1 (BCL2-related) | AI275690 | 0.000782 | 4170 | MCL1 | 1q21 |
| 240432_x_at | Transcribed locus. weakly similar to XP_548293.1 similar to Flotillin-2 (Reggie-1) (REG-1) [Canis familiaris] | AI333006 | 0.000816 | --- | --- | --- |
| 202910_s_at | CD97 antigen | NM_001784 | 0.000951 | 976 | CD97 | 19p13 |
| 238652_at | Amine oxidase (flavin containing) domain 2 | AW419203 | 0.000968 | --- | --- | --- |
| 225831_at | leucine zipper protein 1 | AW016830 | 0.000988 | 7798 | LUZP1 | 1p36 |
| 208810_at | DnaJ (Hsp40) homolog subfamily B. member 6 | AF080569 | 0.001018 | 10049 | DNAJB6 | 7q36.3 |
| 1559425_at | Protein kinase C eta | AL512701 | 0.00102 | --- | --- | --- |
| 209898_x_at | intersectin 2 | U61167 | 0.001035 | 50618 | ITSN2 | 2pter-p25.1 |
| 202933_s_at | v-yes-1 Yamaguchi sarcoma viral oncogene homolog 1 | NM_005433 | 0.001046 | 7525 | YES1 | 18p11.31-p11.21 |
| 209648_x_at | suppressor of cytokine signaling 5 | AL136896 | 0.001052 | 9655 | SOCS5 | 2p21 |
| 209647_s_at | suppressor of cytokine signaling 5 | AW664421 | 0.001054 | 9655 | SOCS5 | 2p21 |
| 208127_s_at | suppressor of cytokine signaling 5 | NM_014011 | 0.001058 | 9655 | SOCS5 | 2p21 |
| 231990_at | ubiquitin specific protease 15 | AK023703 | 0.00107 | 9958 | USP15 | 12q14 |
| 213666_at | septin 6 | AK026589 | 0.001136 | 23157 | SEPT6 | Xq24 |
| 239379_at | CDNA FLJ13876 fis. clone THYRO1001401 | AW449624 | 0.001164 | 9208 | LRRFIP1 | 2q37.3 |
| 224829_at | cytoplasmic polyadenylation element binding protein 4 | AA772278 | 0.001247 | 80315 | CPEB4 | 5q21 |
| 220966_x_at | actin related protein 2/3 complex. subunit 5-like | NM_030978 | 0.001249 | 81873 | ARPC5L | 9q33.3 |
| 1561167_at | Ets variant gene 6 (TEL oncogene) | AF147300 | 0.001285 | --- | --- | --- |
| 243465_at | Hypothetical protein FLJ11000 | AI033097 | 0.001303 | --- | --- | --- |
| 240665_at | CUG triplet repeat RNA binding protein 2 | R40515 | 0.001349 | --- | --- | --- |
| 224164_at | tropomyosin 3 | AY004867 | 0.001353 | 7170 | TPM3 | 1q21.2 |
| 242814_at | serine (or cysteine) proteinase inhibitor clade B (ovalbumin) member 9 | AI986192 | 0.001434 | 5272 | SERPINB9 | 6p25 |
| 221760_at | Mannosidase alpha. class 1A member 1 | BG287153 | 0.001437 | 4121 | MAN1A1 | 6q22 |
| 223377_x_at | cytokine inducible SH2-containing protein | AF035947 | 0.001456 | 1154 | CISH | 3p21.3 |
| 235483_at | CDNA FLJ30906 fis clone FEBRA2006055 | AA858058 | 0.001474 | --- | --- | --- |
| 208078_s_at | SNF1-like kinase | NM_030751 | 0.001493 | 150094 | SIK1 | 21q22.3 |
| 232949_at | Capping protein (actin filament) muscle Z-line beta | AU146181 | 0.001536 | --- | --- | --- |
| 232083_at | chromosome 20 open reading frame 23 | AB046810 | 0.00157 | 55614 | KIF16B | 20p11.23 |
| 201830_s_at | neuroepithelial cell transforming gene 1 | NM_005863 | 0.001573 | 10276 | NET1 | 10p15 |
| 238488_at | synleurin | BF511602 | 0.001605 | 353281 | hCG_2045899 | 5q12.1 |
| 239740_at | ets variant gene 6 (TEL oncogene) | BF436898 | 0.001637 | 2120 | ETV6 | 12p13 |
| 225557_at | AXIN1 up-regulated 1 | AI091372 | 0.001657 | 64651 | AXUD1 | 3p22 |
| 227309_at | Hypothetical LOC55432 | AI982535 | 0.001777 | 55432 | YOD1 | 1q32.1 |
| 243010_at | musashi homolog 2 (Drosophila) | BE000929 | 0.001806 | 124540 | MSI2 | 17q22 |
| 217478_s_at | major histocompatibility complex class II DM alpha | X76775 | 0.00182 | 3108 | HLA-DMA | 6p21.3 |
| 202932_at | v-yes-1 Yamaguchi sarcoma viral oncogene homolog 1 | NM_005433 | 0.001822 | 7525 | YES1 | 18p11.31-p11.21 |
| 232172_at | hypothetical gene supported by AK125149 | AK023533 | 0.001838 | 401577 | LOC401577 | Xp22.33;Yp11.31 |
| 206099_at | protein kinase C eta | NM_006255 | 0.001907 | 5583 | PRKCH | 14q22-q23 |
| 235536_at | Similar to RIKEN cDNA E030024N20 gene | AI640483 | 0.001949 | 692205 | SNORD89 | 2q11.2 |
| 225177_at | RAB11 family interacting protein 1 (class I) | AA143793 | 0.001957 | 80223 | RAB11FIP1 | 8p11.22 |
| 222408_s_at | yippee-like 5 (Drosophila) | BC000836 | 0.00197 | 51646 | YPEL5 | 2p23.1 |
| 234278_at | gb:AL110230.1 /DB_XREF=gi:5817169 /FEA=mRNA /CNT=1 /TID=Hs.306340.0 /TIER=ConsEnd /STK=0 /UG=Hs.306340 /UG_TITLE=Homo sapiens mRNA; cDNA DKFZp564A0769 (from clone DKFZp564A0769) /DEF=Homo sapiens mRNA; cDNA DKFZp564A0769 (from clone DKFZp564A0769) | AL110230 | 0.001974 | --- | --- | --- |
| 222942_s_at | T-cell lymphoma invasion and metastasis 2 | AI094945 | 0.002204 | 26230 | TIAM2 | 6q25.2 |
| 243819_at | Guanine nucleotide binding protein (G protein)gamma 2 | AU146329 | 0.002243 | --- | --- | --- |
| 220046_s_at | cyclin L1 | NM_020307 | 0.002249 | 57018 | CCNL1 | 3q25.32 |
| 202364_at | MAX interactor 1 | NM_005962 | 0.002259 | 4601 | MXI1 | 10q24-q25 |
| 243213_at | signal transducer and activator of transcription 3 (acute-phase response factor) | BF508977 | 0.002284 | 6774 | STAT3 | 17q21.31 |
| 222142_at | cylindromatosis (turban tumor syndrome) | AK024212 | 0.002285 | 1540 | CYLD | 16q12.1 |
| 224862_at | Guanine nucleotide binding protein (G protein) q polypeptide | BF969428 | 0.002287 | 2776 | GNAQ | 9q21 |
| 223085_at | ring finger protein 19 | AB029316 | 0.002293 | 25897 | RNF19A | 8q22 |
| 229975_at | Transcribed locus | AI826437 | 0.002324 | 658 | BMPR1B | 4q22-q24 |
| 228549_at | gb:AI491983 /DB_XREF=gi:4392986 /DB_XREF=to07e03.x1 /CLONE=IMAGE:2178364 /FEA=EST /CNT=28 /TID=Hs.322704.0 /TIER=Stack /STK=14 /UG=Hs.322704 /UG_TITLE=ESTs | AI491983 | 0.002351 | 9725 | TMEM63A | 1q42.12 |
| 1555411_a_at | cyclin L1 | AF367476 | 0.002372 | 57018 | CCNL1 | 3q25.32 |
| 201829_at | neuroepithelial cell transforming gene 1 | AW263232 | 0.002374 | 10276 | NET1 | 10p15 |
| 203799_at | CD302 antigen | NM_014880 | 0.002431 | 9936 | CD302 | 2q24.2 |
| 202861_at | period homolog 1 (Drosophila) | NM_002616 | 0.002435 | 5187 | PER1 | 17p13.1-p12 |
| 229593_at | H2A histone family member Y | BE503981 | 0.002505 | --- | --- | --- |
| 229718_at | Hypothetical gene CG018 | BF448287 | 0.002529 | 90634 | N4BP2L1 | 13q12-q13 |
| 218611_at | immediate early response 5 | NM_016545 | 0.002661 | 51278 | IER5 | 1q25.3 |
| 212079_s_at | myeloid/lymphoid or mixed-lineage leukemia (trithorax homolog Drosophila) | AA715041 | 0.002668 | 4297 | MLL | 11q23 |
| 229419_at | F-box and WD-40 domain protein 7 (archipelago homolog Drosophila) | BF222826 | 0.002682 | 55294 | FBXW7 | 4q31.3 |
| 222729_at | F-box and WD-40 domain protein 7 (archipelago homolog. Drosophila) | BE551877 | 0.002708 | 55294 | FBXW7 | 4q31.3 |
| 206135_at | suppression of tumorigenicity 18 (breast carcinoma) (zinc finger protein) | NM_014682 | 0.002717 | 9705 | ST18 | 8q11.23 |
| 226915_s_at | actin related protein 2/3 complex subunit 5-like | AU158936 | 0.002719 | 81873 | ARPC5L | 9q33.3 |
| 1555847_a_at | hypothetical protein LOC284454 | BU617052 | 0.002733 | 284454 | LOC284454 | 19p13.13 |
| 1555960_at | Histidine triad nucleotide binding protein 1 | AK054976 | 0.002793 | 3094 | HINT1 | 5q31.2 |
| 1570588_at | Myosin phosphatase-Rho interacting protein | AI859267 | 0.002833 | --- | --- | --- |
| 236656_s_at | Full length insert cDNA YI37C01 | AW014647 | 0.002834 | 100130506 | LOC100130506 | --- |
| 222139_at | KIAA1466 gene | AI765383 | 0.002922 | 57612 | KIAA1466 | 7q33 |
| 225950_at | Transcribed locus. weakly similar to XP_496299.1 hypothetical protein LOC148206 [Homo sapiens] | AV703731 | 0.003021 | --- | --- | --- |
| 202820_at | aryl hydrocarbon receptor | NM_001621 | 0.003105 | 196 | AHR | 7p15 |
| 243178_at | gb:AW969703 /DB_XREF=gi:8159547 /DB_XREF=EST381780 /FEA=EST /CNT=5 /TID=Hs.142074.0 /TIER=ConsEnd /STK=0 /UG=Hs.142074 /UG_TITLE=ESTs. Weakly similar to ALU1_HUMAN ALU SUBFAMILY J SEQUENCE CONTAMINATION WARNING ENTRY (H.sapiens) | AW969703 | 0.003108 | --- | --- | --- |
| 202464_s_at | 6-phosphofructo-2-kinase/fructose-2.6-biphosphatase 3 | NM_004566 | 0.003119 | 5209 | PFKFB3 | 10p14-p15 |
| 230348_at | LATS large tumor suppressor homolog 2 (Drosophila) | AI745254 | 0.003154 | 26524 | LATS2 | 13q11-q12 |
| 232809_s_at | Fms-related tyrosine kinase 1 (vascular endothelial growth factor/vascular permeability factor receptor) | AK026896 | 0.00317 | 2321 | FLT1 | 13q12 |
| 227697_at | suppressor of cytokine signaling 3 | AI244908 | 0.003196 | 9021 | SOCS3 | 17q25.3 |
| 219144_at | dual specificity phosphatase 26 (putative) | NM_024025 | 0.003217 | 78986 | DUSP26 | 8p12 |
| 218964_at | AT rich interactive domain 3B (BRIGHT- like) | NM_006465 | 0.003225 | 10620 | ARID3B | 15q24 |
| 237703_at | Ribosomal protein S10 | AI655394 | 0.003238 | --- | --- | --- |
| 208735_s_at | CTD (carboxy-terminal domain. RNA polymerase II. polypeptide A) small phosphatase 2 | AF022231 | 0.003284 | 10106 | CTDSP2 | 12q13-q15 |
| 206380_s_at | properdin P factor. complement | NM_002621 | 0.003288 | 5199 | CFP | Xp11.3-p11.23 |
| 224828_at | cytoplasmic polyadenylation element binding protein 4 | AV704132 | 0.003367 | 80315 | CPEB4 | 5q21 |
| 242579_at | Transcribed locus | AA935461 | 0.003393 | 658 | BMPR1B | 4q22-q24 |
| 223741_s_at | tweety homolog 2 (Drosophila) | BC004233 | 0.003478 | 94015 | TTYH2 | 17q25.1 |
| 230123_at | adaptin-ear-binding coat-associated protein 2 | AI608836 | 0.003519 | 55707 | NECAP2 | 1p36.13 |
| 218751_s_at | F-box and WD-40 domain protein 7 (archipelago homolog Drosophila) | NM_018315 | 0.003562 | 55294 | FBXW7 | 4q31.3 |
| 203543_s_at | Kruppel-like factor 9 | NM_001206 | 0.003581 | 687 | KLF9 | 9q13 |
| 225764_at | ets variant gene 6 (TEL oncogene) | AI762695 | 0.003642 | 2120 | ETV6 | 12p13 |
| 204334_at | Kruppel-like factor 7 (ubiquitous) | AA488672 | 0.003643 | 8609 | KLF7 | 2q32 |
| 209238_at | syntaxin 3A | BE966922 | 0.003654 | 6809 | STX3 | 11q12.1 |
| 227353_at | gb:BE671663 /DB_XREF=gi:10032204 /DB_XREF=7a55g10.x1 /CLONE=IMAGE:3222690 /FEA=EST /CNT=37 /TID=Hs.15284.0 /TIER=Stack /STK=27 /UG=Hs.15284 /UG_TITLE=ESTs | BE671663 | 0.00367 | 147138 | TMC8 | 17q25.3 |
| 209780_at | putative homeodomain transcription factor 2 | AL136883 | 0.003677 | 57157 | PHTF2 | 7q11.23-q21 |
| 239486_at | Transcribed locus | BG111636 | 0.003811 | --- | --- | --- |
| 212463_at | CD59 antigen p18-20 (antigen identified by monoclonal antibodies 16.3A5. EJ16. EJ30. EL32 and G344) | BE379006 | 0.003813 | 966 | CD59 | 11p13 |
| 202083_s_at | SEC14-like 1 (S. cerevisiae) | AI017770 | 0.003874 | 6397 | SEC14L1 | 17q25.1-q25.2 |
| 241756_at | SWI/SNF related. matrix associated. actin dependent regulator of chromatin. subfamily a. member 2 | T51136 | 0.003911 | --- | --- | --- |
| 223533_at | factor for adipocyte differentiation 158 | AL136919 | 0.003931 | 84230 | LRRC8C | 1p22.2 |
| 210001_s_at | suppressor of cytokine signaling 1 | AB005043 | 0.00403 | 8651 | SOCS1 | 16p13.13 |
| 218862_at | ankyrin repeat and SOCS box-containing 13 | NM_024701 | 0.00406 | 79754 | ASB13 | 10p15.1 |
| 210233_at | interleukin 1 receptor accessory protein | AF167343 | 0.004092 | 3556 | IL1RAP | 3q28 |
| 1557813_at | Single-stranded DNA binding protein 2 | BF724621 | 0.004209 | --- | --- | --- |
| 213359_at | Heterogeneous nuclear ribonucleoprotein D (AU-rich element RNA binding protein 1.37kDa) | W74620 | 0.004299 | 3184 | HNRNPD | 4q21.1-q21.2 |
| 242268_at | CUG triplet repeat RNA binding protein 2 | BE157991 | 0.004326 | 10659 | CUGBP2 | 10p13 |
| 236489_at | Transcribed locus | AI282097 | 0.004334 | --- | --- | --- |
| 212225_at | putative translation initiation factor | AL516854 | 0.004371 | 10209 | EIF1 | 17q21.2 |
| 1554876_a_at | S100 calcium binding protein. zeta | AF437876 | 0.004483 | 170591 | S100Z | 5q13.3 |
| 209545_s_at | receptor-interacting serine-threonine kinase 2 | AF064824 | 0.004495 | 8767 | RIPK2 | 8q21 |
| 203069_at | gb:NM_014849.1 /DB_XREF=gi:7662271 /GEN=KIAA0736 /FEA=FLmRNA /CNT=151 /TID=Hs.7979.0 /TIER=FL+Stack /STK=26 /UG=Hs.7979 /LL=9900 /DEF=Homo sapiens KIAA0736 gene product (KIAA0736). mRNA. /PROD=KIAA0736 gene product /FL=gb:AB018279.1 gb:NM_014849.1 | NM_014849 | 0.004507 | 9900 | SV2A | 1q21.2 |
| 242431_at | Ariadne homolog. ubiquitin-conjugating enzyme E2 binding protein. 1 (Drosophila) | AI656728 | 0.004621 | --- | --- | --- |
| 229307_at | ankyrin repeat domain 28 | N32051 | 0.004639 | 23243 | ANKRD28 | 3p24.3 |
| 223218_s_at | nuclear factor of kappa light polypeptide gene enhancer in B-cells inhibitor zeta | AB037925 | 0.004642 | 64332 | NFKBIZ | 3p12-q12 |
| 1560754_at | chemokine-like factor super family 7 | AL832450 | 0.004674 | 112616 | CMTM7 | 3p22.3 |
| 209436_at | spondin 1 extracellular matrix protein | AB018305 | 0.004692 | 10418 | SPON1 | 11p15.2 |
| 240960_at | Moesin | AA713522 | 0.004698 | --- | --- | --- |
| 223262_s_at | FGFR1 oncogene partner 2 | AI738434 | 0.004717 | 26127 | FGFR1OP2 | 12p11.23 |
| 235959_at | AT rich interactive domain 4B (RBP1- like) | AI424238 | 0.00479 | --- | --- | --- |
| 242064_at | sidekick homolog 2 (chicken) | N23651 | 0.004794 | 54549 | SDK2 | 17q25.1 |
| 216985_s_at | syntaxin 3A | AJ002077 | 0.004857 | 6809 | STX3 | 11q12.1 |
| 218012_at | TSPY-like 2 | NM_022117 | 0.004873 | 64061 | TSPYL2 | Xp11.2 |
| 219938_s_at | proline-serine-threonine phosphatase interacting protein 2 | NM_024430 | 0.0049 | 9050 | PSTPIP2 | 18q12 |
| 209732_at | C-type lectin domain family 2 member B | BC005254 | 0.004942 | 9976 | CLEC2B | 12p13-p12 |
| 235142_at | zinc finger and BTB domain containing 8 | AW006067 | 0.004986 | 653121 /// 728116 | RP1-27O5.1 /// ZBTB8 | 1p35.1 |
| 201670_s_at | myristoylated alanine-rich protein kinase C substrate | M68956 | 0.005054 | 4082 | MARCKS | 6q22.2 |
| 229483_at | Ubiquitin-conjugating enzyme E2H (UBC8 homolog. yeast) | AA760738 | 0.005081 | --- | --- | --- |
| 212515_s_at | DEAD (Asp-Glu-Ala-Asp) box polypeptide 3. X-linked | BG492602 | 0.005083 | 1654 | DDX3X | Xp11.3-p11.23 |
| 238534_at | CDNA FLJ13876 fis. clone THYRO1001401 | AA262583 | 0.005207 | 9208 | LRRFIP1 | 2q37.3 |
| 228471_at | Hypothetical protein DKFZp434D2328 | AA744636 | 0.00527 | --- | --- | --- |
| 1554352_s_at | c-myc promoter binding protein | BC041706 | 0.0053 | 10260 | DENND4A | 15q22.31 |
| 228007_at | MRNA; cDNA DKFZp434O0921 (from clone DKFZp434O0921) | AL133101 | 0.005308 | 387119 | C6orf204 | 6q22 |
| 226095_s_at | hypothetical protein LOC146517 | AW138861 | 0.005339 | 342371 | ATXN1L | 16q22.3 |
| 229625_at | Guanylate binding protein 5 | BG545653 | 0.005368 | 115362 | GBP5 | 1p22.2 |
| 241150_at | Spectrin. alpha. non-erythrocytic 1 (alpha-fodrin) | AI668588 | 0.005427 | 6709 | SPTAN1 | 9q33-q34 |
| 235317_at | hypothetical protein LOC284454 | AW975045 | 0.005438 | 284454 | LOC284454 | 19p13.13 |
| 206488_s_at | CD36 antigen (collagen type I receptorthrombospondin receptor) | NM_000072 | 0.00545 | 948 | CD36 | 7q11.2 |
| 228702_at | hypothetical protein FLJ43663 | AL569506 | 0.005473 | 378805 | FLJ43663 | 7q32.3 |
| 239835_at | T-cell activation kelch repeat protein | AA669114 | 0.005494 | 84541 | KBTBD8 | 3p14 |
| 1554661_s_at | hypothetical protein FLJ32001 | BC036200 | 0.005498 | 163882 | C1orf71 | 1q44 |
| 239721_at | Ubiquitin-conjugating enzyme E2H (UBC8 homolog. yeast) | AI280328 | 0.00551 | --- | --- | --- |
| 201566_x_at | inhibitor of DNA binding 2. dominant negative helix-loop-helix protein /// inhibitor of DNA binding 2B. dominant negative helix-loop-helix protein | D13891 | 0.00552 | 3398 | ID2 | 2p25 |
| 1568830_at | Interleukin-1 receptor-associated kinase 3 | BC029493 | 0.005527 | 11213 | IRAK3 | 12q14.3 |
| 215147_at | CUG triplet repeat RNA binding protein 2 | AF007147 | 0.005634 | --- | --- | --- |
| 240282_at | WD repeat domain 1 | AW770902 | 0.005809 | 9948 | WDR1 | 4p16.1 |
| 223708_at | C1q and tumor necrosis factor related protein 4 | AF329838 | 0.005848 | 114900 | C1QTNF4 | 11q11 |
| 234151_at | CUG triplet repeat. RNA binding protein 2 | AK024629 | 0.005997 | --- | --- | --- |
| 239272_at | matrix metalloproteinase 28 | AI927208 | 0.006003 | 79148 | MMP28 | 17q11-q21.1 |
| 244061_at | Rho GTPase activating protein 15 | AI510829 | 0.006388 | --- | --- | --- |
| 242320_at | Forkhead box O3A | AI435586 | 0.006416 | --- | --- | --- |
| 223101_s_at | actin related protein 2/3 complex subunit 5-like | BC000018 | 0.006427 | 81873 | ARPC5L | 9q33.3 |
| 206715_at | transcription factor EC | NM_012252 | 0.006458 | 22797 | TFEC | 7q31.2 |
| 215671_at | Phosphodiesterase 4B cAMP-specific (phosphodiesterase E4 dunce homolog. Drosophila) | AU144792 | 0.006551 | 5142 | PDE4B | 1p31 |
| 203542_s_at | Kruppel-like factor 9 | AI690205 | 0.006567 | 687 | KLF9 | 9q13 |
| 239614_x_at | Glutaminase | AW173003 | 0.006858 | --- | --- | --- |
| 223669_at | hemogen | AF130060 | 0.00689 | 55363 | HEMGN | 9q22.33 |
| 217966_s_at | chromosome 1 open reading frame 24 | NM_022083 | 0.006972 | 116496 | FAM129A | 1q25 |
| 237018_at | A kinase (PRKA) anchor protein 13 | AI051967 | 0.006992 | --- | --- | --- |
| 227223_at | RNA-binding region (RNP1. RRM) containing 2 | BE466173 | 0.007018 | 9584 | RBM39 | 20q11.22 |
| 235716_at | Transformer-2 alpha | AW157450 | 0.007125 | --- | --- | --- |
| 243888_at | Transcribed locus. weakly similar to XP_530365.1 LOC458681 [Pan troglodytes] | AI610684 | 0.007142 | --- | --- | --- |
| 1568665_at | ring finger protein 103 | BC022477 | 0.007251 | 7844 | RNF103 | 2p11.2 |
| 230802_at | gb:AI761947 /DB_XREF=gi:5177614 /DB_XREF=wh50b09.x1 /CLONE=IMAGE:2384153 /FEA=EST /CNT=11 /TID=Hs.131587.0 /TIER=Stack /STK=8 /UG=Hs.131587 /UG_TITLE=ESTs. Weakly similar to Y053_HUMAN HYPOTHETICAL PROTEIN KIAA0053 (H.sapiens) | AI761947 | 0.007319 | 83478 | ARHGAP24 | 4q21.23-q21.3 |
| 208811_s_at | DnaJ (Hsp40) homolog subfamily B. member 6 | AF080569 | 0.007401 | 10049 | DNAJB6 | 7q36.3 |
| 209475_at | ubiquitin specific protease 15 | AF106069 | 0.007506 | 9958 | USP15 | 12q14 |
| 242362_at | Cullin 3 | AI797788 | 0.007532 | --- | --- | --- |
| 231109_at | CUG triplet repeat. RNA binding protein 2 | R44974 | 0.007622 | --- | --- | --- |
| 1568627_at | KIAA1387 protein | BC032531 | 0.007638 | 57223 | SMEK2 | 2p16.1 |
| 242832_at | period homolog 1 (Drosophila) | AI743776 | 0.007717 | 5187 | PER1 | 17p13.1-p12 |
| 1569477_at | Forkhead box O3A | BC025999 | 0.007781 | --- | --- | --- |
| 201565_s_at | inhibitor of DNA binding 2. dominant negative helix-loop-helix protein | NM_002166 | 0.007844 | 3398 | ID2 | 2p25 |
| 223670_s_at | hemogen | AF322875 | 0.007901 | 55363 | HEMGN | 9q22.33 |
| 235912_at | Chr2 synaptotagmin | BE552155 | 0.007913 | --- | --- | --- |
| 201445_at | calponin 3 acidic | NM_001839 | 0.007917 | 1266 | CNN3 | 1p22-p21 |
| 214873_at | hypothetical protein DKFZp434O0213 | AL137651 | 0.008085 | 91355 | LRP5L | 22q11.23 |
| 201029_s_at | CD99 antigen | NM_002414 | 0.008086 | 4267 | CD99 | Xp22.32; Yp11.3 |
| 238774_at | Hypothetical protein LOC284058 | AW960454 | 0.008122 | --- | --- | --- |
| 233647_s_at | cytidine and dCMP deaminase domain containing 1 | AL138875 | 0.008215 | 81602 | CDADC1 | 13q14.2 |
| 217682_at | PRO0149 protein | AW503390 | 0.008335 | --- | --- | --- |
| 218319_at | pellino homolog 1 (Drosophila) | NM_020651 | 0.008346 | 57162 | PELI1 | 2p13.3 |
| 230203_at | LOC440918 | AI866583 | 0.008555 | 440918 | FLJ46875 | 2q24.1 |
| 242868_at | Endothelial PAS domain protein 1 | T70087 | 0.008653 | --- | --- | --- |
| 222326_at | Phosphodiesterase 4B cAMP-specific (phosphodiesterase E4 dunce homolog. Drosophila) | AW973834 | 0.008847 | --- | --- | --- |
| 203056_s_at | PR domain containing 2 with ZNF domain | AI681013 | 0.008869 | 7799 | PRDM2 | 1p36.21 |
| 239635_at | RNA binding motif protein 14 /// hypothetical protein MGC15912 | BF510708 | 0.009061 | 10432 | RBM14 | 11q13.1 |
| 201170_s_at | basic helix-loop-helix domain containing. class B. 2 | NM_003670 | 0.00914 | 8553 | BHLHE40 | 3p26 |
| 206302_s_at | nudix (nucleoside diphosphate linked moiety X)-type motif 4 | NM_019094 | 0.009144 | 11163 /// 440672 | NUDT4 /// NUDT4P1 | 12q21 /// 1q21.1 |
| 37028_at | protein phosphatase 1. regulatory (inhibitor) subunit 15A | U83981 | 0.009205 | 23645 | PPP1R15A | 19q13.2 |
| 232304_at | Pellino homolog 1 (Drosophila) | AK026714 | 0.009216 | 57162 | PELI1 | 2p13.3 |
| 237239_at | gb:AW183655 /DB_XREF=gi:6452169 /DB_XREF=xj86c06.x1 /CLONE=IMAGE:2664106 /FEA=EST /CNT=5 /TID=Hs.189079.0 /TIER=ConsEnd /STK=5 /UG=Hs.189079 /UG_TITLE=ESTs | AW183655 | 0.00931 | --- | --- | --- |
| 226267_at | jun dimerization protein 2 | AA716425 | 0.009313 | 122953 | JDP2 | 14q24.3 |
| 242051_at | Transcribed locus | AI695695 | 0.009323 | --- | --- | --- |
| 244197_x_at | CCR4-NOT transcription complex. subunit 2 | AI859990 | 0.009358 | --- | --- | --- |
| 200983_x_at | CD59 antigen p18-20 (antigen identified by monoclonal antibodies 16.3A5. EJ16. EJ30. EL32 and G344) | BF983379 | 0.00962 | 966 | CD59 | 11p13 |
| 223263_s_at | FGFR1 oncogene partner 2 | AF161472 | 0.00966 | 26127 | FGFR1OP2 | 12p11.23 |
| 239317_at | gb:BG484601 /DB_XREF=gi:13416880 /DB_XREF=602505772F1 /CLONE=IMAGE:4619369 /FEA=EST /CNT=8 /TID=Hs.282899.0 /TIER=ConsEnd /STK=0 /UG=Hs.282899 /UG_TITLE=ESTs | BG484601 | 0.009682 | --- | --- | --- |
| 244110_at | gb:BE669782 /DB_XREF=gi:10030323 /DB_XREF=7e35h03.x1 /CLONE=IMAGE:3284501 /FEA=EST /CNT=3 /TID=Hs.283407.0 /TIER=ConsEnd /STK=3 /UG=Hs.283407 /UG_TITLE=ESTs | BE669782 | 0.009695 | 4297 | MLL | 11q23 |
| 221223_x_at | cytokine inducible SH2-containing protein | NM_013324 | 0.009796 | 1154 | CISH | 3p21.3 |
| 1558111_at | Muscleblind-like (Drosophila) | AL562860 | 0.009819 | 4154 | MBNL1 | 3q25 |
| 208632_at | ring finger protein 10 | AL578551 | 0.009882 | 9921 | RNF10 | 12q24.31 |
| 211302_s_at | phosphodiesterase 4B cAMP-specific (phosphodiesterase E4 dunce homolog. Drosophila) | L20966 | 0.009974 | 5142 | PDE4B | 1p31 |
| 239597_at | PABP1-dependent poly A-specific ribonuclease subunit PAN3 | AA993566 | 0.009985 | --- | --- | --- |
| 230133_at | Membrane associated DNA binding protein | BF972355 | 0.010057 | 54542 | RC3H2 | 9q34 |
| 200895_s_at | FK506 binding protein 4. 59kDa | NM_002014 | 0.010066 | 2288 | FKBP4 | 12p13.33 |
| 241865_at | Guanine nucleotide binding protein (G protein)beta polypeptide 1 | AI056689 | 0.010308 | --- | --- | --- |
| 243286_at | Cullin 1 | AA682674 | 0.010382 | --- | --- | --- |
| 244358_at | gb:AW372457 /DB_XREF=gi:6877020 /DB_XREF=PM0-BT0340-231199-001-h11 /FEA=EST /CNT=7 /TID=Hs.212084.0 /TIER=ConsEnd /STK=0 /UG=Hs.212084 /UG_TITLE=ESTs | AW372457 | 0.010535 | --- | --- | --- |
| 202284_s_at | cyclin-dependent kinase inhibitor 1A (p21. Cip1) | NM_000389 | 0.010542 | 1026 | CDKN1A | 6p21.2 |
| 203322_at | KIAA0863 protein | AU145934 | 0.01072 | 22850 | ADNP2 | 18q23 |
| 239923_at | Forkhead box O3A | AI056872 | 0.01074 | --- | --- | --- |
| 217764_s_at | RAB31 member RAS oncogene family | AF183421 | 0.010757 | 11031 | RAB31 | 18p11.3 |
| 244677_at | gb:AA416756 /DB_XREF=gi:2077710 /DB_XREF=zu08h08.s1 /CLONE=IMAGE:731295 /FEA=EST /CNT=6 /TID=Hs.161051.0 /TIER=ConsEnd /STK=1 /UG=Hs.161051 /UG_TITLE=ESTs. Moderately similar to ALU6_HUMAN ALU SUBFAMILY SP SEQUENCE CONTAMINATION WARNING ENTRY (H.sapiens) | AA416756 | 0.010761 | --- | --- | --- |
| 209339_at | seven in absentia homolog 2 (Drosophila) | U76248 | 0.01082 | 6478 | SIAH2 | 3q25 |
| 235556_at | Transcribed locus. weakly similar to NP_703324.1 Plasmodium falciparum 3D7 MAL1P3.06 gene | AL037805 | 0.010829 | --- | --- | --- |
| 228180_at | Smu-1 suppressor of mec-8 and unc-52 homolog (C. elegans) | AA805653 | 0.01101 | --- | --- | --- |
| 233813_at | protein phosphatase 1. regulatory (inhibitor) subunit 16B | AK026900 | 0.011017 | 26051 | PPP1R16B | 20q11.23 |
| 206036_s_at | v-rel reticuloendotheliosis viral oncogene homolog (avian) | NM_002908 | 0.011028 | 5966 | REL | 2p13-p12 |
| 232213_at | Pellino homolog 1 (Drosophila) | AU147506 | 0.011056 | 57162 | PELI1 | 2p13.3 |
| 222669_s_at | Shwachman-Bodian-Diamond syndrome | AK001779 | 0.011219 | 155370 /// 51119 | SBDS /// SBDSP | 7q11.21 /// 7q11.23 |
| 226370_at | kelch-like 15 (Drosophila) | BG149487 | 0.011252 | 80311 | KLHL15 | Xp22.1-p21 |
| 223527_s_at | cytidine and dCMP deaminase domain containing 1 | AL138875 | 0.011264 | 81602 | CDADC1 | 13q14.2 |
| 223746_at | serine/threonine kinase 4 | BC005231 | 0.011266 | 6789 | STK4 | 20q11.2-q13.2 |
| 214696_at | hypothetical protein MGC14376 | AF070569 | 0.011351 | 84981 | C17orf91 | 17p13.3 |
| 1557814_a_at | Single-stranded DNA binding protein 2 | BF724621 | 0.011538 | --- | --- | --- |
| 213931_at | inhibitor of DNA binding 2. dominant negative helix-loop-helix protein | AI819238 | 0.011546 | 3398 /// 84099 | ID2 /// ID2B | 2p25 /// 3p14.2 |
| 1564424_at | Chr2 synaptotagmin | BC035983 | 0.011554 | --- | --- | --- |
| 217967_s_at | chromosome 1 open reading frame 24 | AF288391 | 0.011686 | 116496 | FAM129A | 1q25 |
| 217741_s_at | zinc finger A20 domain containing 2 | AW471220 | 0.011733 | 7763 | ZFAND5 | 9q13-q21 |
| 225660_at | sema domain transmembrane domain (TM) and cytoplasmic domain (semaphorin) 6A | W92748 | 0.011896 | 57556 | SEMA6A | 5q23.1 |
| 238736_at | REV3-like. catalytic subunit of DNA polymerase zeta (yeast) | AA805939 | 0.011942 | 5980 | REV3L | 6q21 |
| 36711_at | v-maf musculoaponeurotic fibrosarcoma oncogene homolog F (avian) | AL021977 | 0.011998 | 23764 | MAFF | 22q13.1 |
| 202973_x_at | family with sequence similarity 13. member A1 | NM_014883 | 0.012322 | 10144 | FAM13A1 | 4q22.1 |
| 229649_at | neurexin 3 | AI129949 | 0.01233 | 9369 | NRXN3 | 14q31 |
| 218468_s_at | gremlin 1 homolog. cysteine knot superfamily (Xenopus laevis) | AF154054 | 0.01237 | 26585 | GREM1 | 15q13-q15 |
| 238581_at | Guanylate binding protein 5 | BG271923 | 0.012381 | 115362 | GBP5 | 1p22.2 |
| 239232_at | Musashi homolog 2 (Drosophila) | AA521410 | 0.01253 | 124540 | MSI2 | 17q22 |
| 1568943_at | Inositol polyphosphate-5-phosphatase 145kDa | BC027960 | 0.012572 | 3635 | INPP5D | 2q37.1 |
| 214683_s_at | CDC-like kinase 1 | AI251890 | 0.012615 | 1195 | CLK1 | 2q33 |
| 244010_at | B melanoma antigen family. member 4 | AI057455 | 0.01262 | --- | --- | --- |
| 38671_at | plexin D1 | AB014520 | 0.012686 | 23129 | PLXND1 | 3q21.3 |
| 243827_at | Testis expressed sequence 27 | AL038125 | 0.01269 | --- | --- | --- |
| 240539_at | Full length insert cDNA clone ZD73H04 | AI684551 | 0.012732 | --- | --- | --- |
| 230599_at | Ring finger protein 19 | AI681558 | 0.012989 | --- | --- | --- |
| 214172_x_at | RYK receptor-like tyrosine kinase | BG032035 | 0.013293 | 6259 | RYK | 3q22 |
| 219449_s_at | hypothetical protein FLJ20533 | NM_017866 | 0.013337 | 54968 | TMEM70 | 8q21.11 |
| 218086_at | neural proliferation. differentiation and control. 1 | NM_015392 | 0.01343 | 56654 | NPDC1 | 9q34.3 |
| 201669_s_at | myristoylated alanine-rich protein kinase C substrate | NM_002356 | 0.013573 | 4082 | MARCKS | 6q22.2 |
| 214657_s_at | Trophoblast-derived noncoding RNA | AU134977 | 0.013624 | 283131 | NCRNA00084 | 11q13.1 |
| 212842_x_at | RAN binding protein 2-like 1 /// similar to Ran-binding protein 2 /// similar to RAN-binding protein 2-like 1 | AL043571 | 0.013645 | 285190 /// 727851 /// 729540 /// 84220 | RGPD4 /// RGPD5 /// RGPD6 /// RGPD8 | 2q12.3 /// 2q13 |
| 238509_at | Cullin 1 | AI628926 | 0.013734 | 8454 | CUL1 | 7q36.1 |
| 216997_x_at | transducin-like enhancer of split 4 (E(sp1) homolog. Drosophila) | AL358975 | 0.01382 | 7091 | TLE4 | 9q21.31 |
| 243256_at | MAP kinase interacting serine/threonine kinase 1 | AW796364 | 0.013895 | 8569 | MKNK1 | 1p33 |
| 227373_at | hypothetical protein LOC146517 | AW299653 | 0.01395 | 342371 | ATXN1L | 16q22.3 |
| 223380_s_at | LATS large tumor suppressor homolog 2 (Drosophila) | AF207547 | 0.01413 | 26524 | LATS2 | 13q11-q12 |
| 201502_s_at | nuclear factor of kappa light polypeptide gene enhancer in B-cells inhibitor alpha | AI078167 | 0.014198 | 4792 | NFKBIA | 14q13 |
| 240008_at | AT rich interactive domain 1B (SWI1-like) | AI955765 | 0.014203 | --- | --- | --- |
| 230077_at | succinate dehydrogenase complex subunit A flavoprotein (Fp) | W90764 | 0.014267 | 220729 /// 255812 /// 6389 /// 727956 | LOC220729 /// SDHA /// SDHALP1 /// SDHALP2 | 3q29 /// 5p15 |
| 213994_s_at | spondin 1. extracellular matrix protein | AI885290 | 0.014283 | 10418 | SPON1 | 11p15.2 |
| 204917_s_at | myeloid/lymphoid or mixed-lineage leukemia (trithorax homolog Drosophila); translocated to 3 | AV756536 | 0.014301 | 4300 | MLLT3 | 9p22 |
| 214500_at | H2A histone family. member Y | AF044286 | 0.014594 | 9555 | H2AFY | 5q31.3-q32 |
| 231199_at | Mak3 homolog (S. cerevisiae) | AA701676 | 0.01484 | --- | --- | --- |
| 220728_at | gb:NM_025120.1 /DB_XREF=gi:13376698 /GEN=FLJ13480 /FEA=FLmRNA /CNT=4 /TID=Hs.288734.0 /TIER=FL /STK=1 /UG=Hs.288734 /LL=80190 /DEF=Homo sapiens hypothetical protein FLJ13480 (FLJ13480). mRNA. /PROD=hypothetical protein FLJ13480 /FL=gb:NM_025120.1 | NM_025120 | 0.014863 | --- | --- | --- |
| 219878_s_at | Kruppel-like factor 13 | NM_015995 | 0.014868 | 51621 | KLF13 | 15q12 |
| 202014_at | protein phosphatase 1 regulatory (inhibitor) subunit 15A | NM_014330 | 0.014922 | 23645 | PPP1R15A | 19q13.2 |
| 244470_at | Ring finger protein 12 | BF063235 | 0.014995 | 51132 | RNF12 | Xq13-q21 |
| 220454_s_at | sema domain transmembrane domain (TM). and cytoplasmic domain (semaphorin) 6A | NM_020796 | 0.01507 | 57556 | SEMA6A | 5q23.1 |
| 224566_at | trophoblast-derived noncoding RNA | AI042152 | 0.015097 | 283131 | NCRNA00084 | 11q13.1 |
| 1553297_a_at | colony stimulating factor 3 receptor (granulocyte) | NM_172313 | 0.015102 | 1441 | CSF3R | 1p35-p34.3 |
| 1563473_at | Protein phosphatase 1 regulatory (inhibitor) subunit 16B | AL833255 | 0.015156 | --- | --- | --- |
| 218311_at | mitogen-activated protein kinase kinase kinase kinase 3 | NM_003618 | 0.015277 | 8491 | MAP4K3 | 2p22.1 |
| 230803_s_at | Rho GTPase activating protein 24 | AI761947 | 0.015293 | 83478 | ARHGAP24 | 4q21.23-q21.3 |
| 239273_s_at | matrix metalloproteinase 28 | AI927208 | 0.015394 | 79148 | MMP28 | 17q11-q21.1 |
| 209316_s_at | HBS1-like (S. cerevisiae) | BC001465 | 0.015419 | 10767 | HBS1L | 6q23-q24 |
| 244447_at | Kruppel-like factor 10 | AW292830 | 0.015463 | --- | --- | --- |
| 1559315_s_at | hypothetical protein LOC144481 | AK054607 | 0.01562 | 144481 | LOC144481 | 12q22 |
| 244753_at | Actinin alpha 4 | BF000430 | 0.015678 | --- | --- | --- |
| 243364_at | gb:AI417756 /DB_XREF=gi:4261260 /DB_XREF=th31a06.x1 /CLONE=IMAGE:2119858 /FEA=EST /CNT=6 /TID=Hs.167236.0 /TIER=ConsEnd /STK=1 /UG=Hs.167236 /UG_TITLE=ESTs. Highly similar to T00065 hypothetical protein KIAA0442 (H.sapiens) | AI417756 | 0.015767 | 26053 | AUTS2 | 7q11.22 |
| 205193_at | v-maf musculoaponeurotic fibrosarcoma oncogene homolog F (avian) | NM_012323 | 0.015894 | 23764 | MAFF | 22q13.1 |
| 228297_at | Calponin 3 acidic | AI807004 | 0.015948 | --- | --- | --- |
| 210054_at | chromosome 4 open reading frame 15 | BC003648 | 0.015986 | 79441 | C4orf15 | 4p16.3 |
| 230134_s_at | membrane associated DNA binding protein | BF972355 | 0.016055 | 54542 | RC3H2 | 9q34 |
| 206303_s_at | nudix (nucleoside diphosphate linked moiety X)-type motif 4 | AF191653 | 0.016083 | 11163 /// 440672 | NUDT4 /// NUDT4P1 | 12q21 /// 1q21.1 |
| 1565651_at | CDNA FLJ33210 fis clone ADRGL2008535 | BI868311 | 0.016201 | 375 | ARF1 | 1q42 |
| 203544_s_at | signal transducing adaptor molecule (SH3 domain and ITAM motif) 1 | NM_003473 | 0.016233 | 8027 | STAM | 10p14-p13 |
| 225912_at | tumor protein p53 inducible nuclear protein 1 | AW341649 | 0.016393 | 94241 | TP53INP1 | 8q22 |
| 205214_at | serine/threonine kinase 17b (apoptosis-inducing) | NM_004226 | 0.016461 | 9262 | STK17B | 2q32.3 |
| 237009_at | CD69 antigen (p60 early T-cell activation antigen) | BF439675 | 0.016549 | --- | --- | --- |
| 233575_s_at | transducin-like enhancer of split 4 (E(sp1) homolog. Drosophila) | AA705845 | 0.016703 | 7091 | TLE4 | 9q21.31 |
| 210999_s_at | growth factor receptor-bound protein 10 | U66065 | 0.016784 | 2887 | GRB10 | 7p12-p11.2 |
| 209304_x_at | growth arrest and DNA-damage-inducible beta | AF087853 | 0.016815 | 4616 | GADD45B | 19p13.3 |
| 214022_s_at | interferon induced transmembrane protein 1 (9-27) | AA749101 | 0.016928 | 8519 | IFITM1 | 11p15.5 |
| 1554089_s_at | Shwachman-Bodian-Diamond syndrome /// Shwachman-Bodian-Diamond syndrome pseudogene | BC010183 | 0.01694 | 155370 /// 51119 | SBDS /// SBDSP | 7q11.21 /// 7q11.23 |
| 215577_at | Ubiquitin-conjugating enzyme E2E 1 (UBC4/5 homolog. yeast) | AU146791 | 0.016959 | --- | --- | --- |
| 203149_at | poliovirus receptor-related 2 (herpesvirus entry mediator B) | NM_002856 | 0.01698 | 5819 | PVRL2 | 19q13.2 |
| 244165_at | gb:AI809511 /DB_XREF=gi:5396077 /DB_XREF=wf30d05.x1 /CLONE=IMAGE:2357097 /FEA=EST /CNT=4 /TID=Hs.204715.0 /TIER=ConsEnd /STK=3 /UG=Hs.204715 /UG_TITLE=ESTs | AI809511 | 0.01721 | 54906 | C10orf18 | 10p15.1 |
| 212443_at | neurobeachin-like 2 | AB011112 | 0.017316 | 23218 | NBEAL2 | 3p21.31 |
| 229228_at | cAMP responsive element binding protein 5 | AI819043 | 0.017569 | 9586 | CREB5 | 7p15.1 |
| 217047_s_at | family with sequence similarity 13 member A1 | AK027138 | 0.01758 | 10144 | FAM13A1 | 4q22.1 |
| 202859_x_at | interleukin 8 | NM_000584 | 0.01789 | 3576 | IL8 | 4q13-q21 |
| 1561195_at | Transmembrane 7 superfamily member 1 (upregulated in kidney) | AL832142 | 0.01789 | --- | --- | --- |
| 205795_at | neurexin 3 | NM_004796 | 0.017908 | 9369 | NRXN3 | 14q31 |
| 230868_at | Hippocampus abundant transcript 1 | BF433103 | 0.018055 | --- | --- | --- |
| 232882_at | Forkhead box O1A (rhabdomyosarcoma) | AA079839 | 0.018099 | --- | --- | --- |
| 1559060_a_at | KIAA1961 gene | BF677986 | 0.018315 | 96459 | FNIP1 | 5q23.3 |
| 217109_at | mucin 4 tracheobronchial | AJ242547 | 0.018328 | 4585 | MUC4 | 3q29 |
| 236216_at | Transcription factor 7-like 2 (T-cell specific. HMG-box) | AA598661 | 0.018513 | --- | --- | --- |
| 209723_at | serine (or cysteine) proteinase inhibitor clade B (ovalbumin) member 9 | BC002538 | 0.018631 | 5272 | SERPINB9 | 6p25 |
| 240383_at | ubiquitin-conjugating enzyme E2D 3 (UBC4/5 homolog. yeast) | AI239832 | 0.01878 | 7323 | UBE2D3 | 4q24 |
| 209555_s_at | CD36 antigen (collagen type I receptorthrombospondin receptor) | M98399 | 0.018872 | 948 | CD36 | 7q11.2 |
| 227622_at | pre-mRNA cleavage complex II protein Pcf11 | AW118175 | 0.01889 | 51585 | PCF11 | 11q13 |
| 201369_s_at | zinc finger protein 36. C3H type-like 2 | NM_006887 | 0.019605 | 678 | ZFP36L2 | 2p22.3-p21 |
| 222420_s_at | ubiquitin-conjugating enzyme E2H (UBC8 homolog yeast) | Z29331 | 0.01966 | 7328 | UBE2H | 7q32 |
| 225108_at | Alkylglycerone phosphate synthase | BF111719 | 0.019692 | 8540 | AGPS | 2q31.2 |
| 238840_at | leucine rich repeat (in FLII) interacting protein 1 | AW082668 | 0.01984 | 9208 | LRRFIP1 | 2q37.3 |
| 244535_at | Forkhead box P1 | AI760944 | 0.019848 | --- | --- | --- |
| 201601_x_at | interferon induced transmembrane protein 1 (9-27) | NM_003641 | 0.019862 | 8519 | IFITM1 | 11p15.5 |
| 1554229_at | adult retina protein | AY174896 | 0.019898 | 153222 | C5orf41 | 5q35.2 |
| 229454_at | BCL2-associated transcription factor 1 | AV717336 | 0.020102 | 9774 | BCLAF1 | 6q22-q23 |
| 228812_at | Transcribed locus. weakly similar to XP_513408.1 similar to origin recognition complex. subunit 1; origin recognition complex. subunit 1. S. cerevisiae. homolog-like; origin recognition complex 1; replication control protein 1; origin recognition complex. subunit 1 (yeast homolog)-like ... [Pan troglodytes] | AI652899 | 0.020869 | --- | --- | --- |
| 206707_x_at | chromosome 6 open reading frame 32 | NM_015864 | 0.02096 | 9750 | FAM65B | 6p22.3-p21.32 |
| 209606_at | pleckstrin homology. Sec7 and coiled-coil domains. binding protein | L06633 | 0.021051 | 9595 | CYTIP | 2q11.2 |
| 201713_s_at | RAN binding protein 2 | D42063 | 0.021312 | 5903 | RANBP2 | 2q12.3 |
| 1569652_at | Myeloid/lymphoid or mixed-lineage leukemia (trithorax homolog Drosophila); translocated to 3 | BC030550 | 0.02143 | 4300 | MLLT3 | 9p22 |
| 203708_at | phosphodiesterase 4B. cAMP-specific (phosphodiesterase E4 dunce homolog. Drosophila) | NM_002600 | 0.021454 | 5142 | PDE4B | 1p31 |
| 225372_at | chromosome 10 open reading frame 54 | AK024449 | 0.021606 | 64115 | C10orf54 | 10q22.1 |
| 236545_at | Protein phosphatase 3 (formerly 2B) catalytic subunit. alpha isoform (calcineurin A alpha) | AA532718 | 0.021682 | --- | --- | --- |
| 239102_s_at | Phosphatidylinositol binding clathrin assembly protein | AW293296 | 0.021793 | --- | --- | --- |
| 242688_at | Thyroid hormone receptor interactor 12 | AI149880 | 0.021941 | --- | --- | --- |
| 212975_at | KIAA0870 protein | AB020677 | 0.022043 | 22898 | DENND3 | 8q24.3 |
| 242712_x_at | gb:BE856960 /DB_XREF=gi:10370511 /DB_XREF=7f71h06.x1 /CLONE=IMAGE:3300155 /FEA=EST /CNT=6 /TID=Hs.167822.0 /TIER=ConsEnd /STK=0 /UG=Hs.167822 /UG_TITLE=ESTs. Highly similar to S58884 Ran-binding protein 2 (H.sapiens) | BE856960 | 0.022125 | 285190 /// 400966 /// 5903 /// 652919 /// 653489 /// 727851 /// 729540 /// 729857 /// 84220 | RANBP2 /// RGPD1 /// RGPD2 /// RGPD3 /// RGPD4 /// RGPD5 /// RGPD6 /// RGPD7 /// RGPD8 | 2p11.2 /// 2q12.3 /// 2q13 |
| 244721_at | tumor protein p53 inducible nuclear protein 1 | AW242000 | 0.022464 | 94241 | TP53INP1 | 8q22 |
| 212590_at | related RAS viral (r-ras) oncogene homolog 2 | AI431643 | 0.022494 | 22800 | RRAS2 | 11p15.2 |
| 211026_s_at | monoglyceride lipase | BC006230 | 0.022508 | 11343 | MGLL | 3q21.3 |
| 236699_at | Muscleblind-like 2 (Drosophila) | AL566294 | 0.022757 | --- | --- | --- |
| 215330_at | Hypothetical protein FLJ43663 | AL049991 | 0.022773 | --- | --- | --- |
| 202499_s_at | solute carrier family 2 (facilitated glucose transporter) member 3 | NM_006931 | 0.022786 | 6515 | SLC2A3 | 12p13.3 |
| 243395_at | Decay accelerating factor for complement (CD55. Cromer blood group system) | AI679555 | 0.022795 | --- | --- | --- |
| 212183_at | nudix (nucleoside diphosphate linked moiety X)-type motif 4 | AW511135 | 0.022813 | 11163 /// 440672 | NUDT4 /// NUDT4P1 | 12q21 /// 1q21.1 |
| 208886_at | H1 histone family member 0 | BC000145 | 0.022871 | 3005 | H1F0 | 22q13.1 |
| 203372_s_at | suppressor of cytokine signaling 2 | AB004903 | 0.022928 | 8835 | SOCS2 | 12q |
| 226632_at | cytoglobin | AL513673 | 0.023317 | 114757 | CYGB | 17q25.3 |
| 239901_at | Hypothetical protein FLJ43663 | BF642798 | 0.023752 | --- | --- | --- |
| 226840_at | H2A histone family. member Y | AW291297 | 0.023858 | 9555 | H2AFY | 5q31.3-q32 |
| 226115_at | ELYS transcription factor-like protein TMBS62 | AI138934 | 0.023861 | 25909 | AHCTF1 | 1q44 |
| 223422_s_at | Rho GTPase activating protein 24 | AI743534 | 0.023905 | 83478 | ARHGAP24 | 4q21.23-q21.3 |
| 235242_at | CDNA FLJ41375 fis clone BRCAN2007700 | BE739287 | 0.023926 | --- | --- | --- |
| 219191_s_at | bridging integrator 2 | NM_016293 | 0.024099 | 51411 | BIN2 | 12q13 |
| 1554026_a_at | Myosin X | BC041694 | 0.024122 | 4651 | MYO10 | 5p15.1-p14.3 |
| 213975_s_at | lysozyme (renal amyloidosis) /// leukocyte immunoglobulin-like receptor subfamily B (with TM and ITIM domains) member 1 | AV711904 | 0.024159 | 4069 | LYZ | 12q15 |
| 203311_s_at | ADP-ribosylation factor 6 | M57763 | 0.024535 | 382 | ARF6 | 14q21.3 |
| 217503_at | gb:AA203487 /DB_XREF=gi:1799460 /DB_XREF=zx53d03.r1 /CLONE=IMAGE:446213 /FEA=EST /CNT=19 /TID=Hs.314363.0 /TIER=ConsEnd /STK=0 /UG=Hs.314363 /UG_TITLE=ESTs | AA203487 | 0.024727 | --- | --- | --- |
| 242403_at | Ubiquitin-conjugating enzyme E2D 3 (UBC4/5 homolog. yeast) | AI459177 | 0.024802 | --- | --- | --- |
| 228665_at | cysteine and tyrosine-rich 1 | AI458003 | 0.024803 | 116159 | CYYR1 | 21q21.2 |
| 203751_x_at | jun D proto-oncogene | AI762296 | 0.025022 | 3727 | JUND | 19p13.2 |
| 240094_at | Hypothetical protein DJ971N18.2 | AL042660 | 0.025134 | --- | --- | --- |
| 242448_at | Mitogen-activated protein kinase kinase kinase kinase 3 | AI800895 | 0.025298 | --- | --- | --- |
| 225789_at | centaurin gamma 3 | BE876194 | 0.025502 | 116988 | AGAP3 | 7q36.1 |
| 207574_s_at | growth arrest and DNA-damage-inducible beta | NM_015675 | 0.025625 | 4616 | GADD45B | 19p13.3 |
| 235847_at | Testis expressed sequence 27 | BF111312 | 0.025655 | --- | --- | --- |
| 1555420_a_at | Kruppel-like factor 7 (ubiquitous) | BC012919 | 0.025733 | 8609 | KLF7 | 2q32 |
| 214574_x_at | leukocyte specific transcript 1 | NM_007161 | 0.025918 | 7940 | LST1 | 6p21.3 |
| 210024_s_at | ubiquitin-conjugating enzyme E2E 3 (UBC4/5 homolog. yeast) | AB017644 | 0.025945 | 10477 | UBE2E3 | 2q32.1 |
| 212592_at | Immunoglobulin J polypeptide linker protein for immunoglobulin alpha and mu polypeptides | AV733266 | 0.025988 | 3512 | IGJ | 4q21 |
| 203725_at | growth arrest and DNA-damage-inducible. alpha | NM_001924 | 0.026152 | 1647 | GADD45A | 1p31.2-p31.1 |
| 1556037_s_at | hedgehog interacting protein | AK098525 | 0.026242 | 64399 | HHIP | 4q28-q32 |
| 209457_at | dual specificity phosphatase 5 | U16996 | 0.026292 | 1847 | DUSP5 | 10q25 |
| 221030_s_at | Rho GTPase activating protein 24 | NM_031305 | 0.026431 | 83478 | ARHGAP24 | 4q21.23-q21.3 |
| 214326_x_at | jun D proto-oncogene | AI339541 | 0.026487 | 3727 | JUND | 19p13.2 |
| 217763_s_at | RAB31 member RAS oncogene family | NM_006868 | 0.026508 | 11031 | RAB31 | 18p11.3 |
| 1561690_at | CDNA clone IMAGE:5303966 partial cds | BC041991 | 0.026514 | --- | --- | --- |
| 224836_at | tumor protein p53 inducible nuclear protein 2 | AL109824 | 0.026655 | 58476 | TP53INP2 | 20q11.22 |
| 214085_x_at | GLI pathogenesis-related 1 (glioma) | AI912583 | 0.026821 | 11010 | GLIPR1 | 12q21.2 |
| 230083_at | Ubiquitin specific protease 53 | AW188464 | 0.026882 | 54532 | USP53 | 4q26 |
| 212235_at | plexin D1 | AL575403 | 0.027013 | 23129 | PLXND1 | 3q21.3 |
| 204198_s_at | runt-related transcription factor 3 | AA541630 | 0.027197 | 864 | RUNX3 | 1p36 |
| 223304_at | solute carrier family 37 (glycerol-3-phosphate transporter) member 3 | AL136583 | 0.027292 | 84255 | SLC37A3 | 7q34 |
| 230747_s_at | CDNA clone IMAGE:3029742 partial cds | AA406435 | 0.027493 | 125488 | TTC39C | 18q11.2 |
| 227923_at | SH3 and multiple ankyrin repeat domains 3 | BF439330 | 0.027578 | 85358 | SHANK3 | 22q13.3 |
| 226275_at | MAX dimerization protein 1 | AI188653 | 0.027596 | 4084 | MXD1 | 2p13-p12 |
| 244646_at | Hypothetical protein FLJ11710 | AW972881 | 0.027644 | --- | --- | --- |
| 1555476_at | iron-responsive element binding protein 2 | BC017880 | 0.027717 | 3658 | IREB2 | 15q25.1 |
| 211582_x_at | leukocyte specific transcript 1 | AF000424 | 0.027792 | 7940 | LST1 | 6p21.3 |
| 201925_s_at | decay accelerating factor for complement (CD55. Cromer blood group system) | NM_000574 | 0.027813 | 1604 | CD55 | 1q32 |
| 225899_x_at | hypothetical protein LOC284701 /// hypothetical gene supported by AK128780 /// FLJ45445 protein /// hypothetical gene supported by AK093729; BX647918 /// similar to hypothetical protein LOC349114 /// hypothetical gene supported by AK097080; AL117642; BC047304; BC054485 | AL040396 | 0.028112 | 388312 /// 402483 /// 643670 /// 728105 | LOC388312 /// LOC643670 /// LOC728105 /// tcag7.907 | 1p36.33 /// 3q29 /// 5q35.3 /// 7q32.1 |
| 236754_at | gb:AI216567 /DB_XREF=gi:3785608 /DB_XREF=qm42b01.x1 /CLONE=IMAGE:1884457 /FEA=EST /CNT=7 /TID=Hs.184840.0 /TIER=ConsEnd /STK=7 /UG=Hs.184840 /UG_TITLE=ESTs | AI216567 | 0.028213 | --- | --- | --- |
| 240038_at | Elongation factor RNA polymerase II 2 | AW057518 | 0.028468 | --- | --- | --- |
| 229574_at | Transformer-2 alpha | AI268231 | 0.02858 | 29896 | TRA2A | 7p15.3 |
| 232175_at | ADP-ribosylation factor 1 | AI972094 | 0.028803 | 375 | ARF1 | 1q42 |
| 227501_at | WD repeat and SOCS box-containing 1 | AI377135 | 0.028966 | --- | --- | --- |
| 217800_s_at | Nedd4 family interacting protein 1 | NM_030571 | 0.028971 | 80762 | NDFIP1 | 5q31.3 |
| 233303_at | Ubiquitin-conjugating enzyme E2D 3 (UBC4/5 homolog. yeast) | AL110175 | 0.029025 | --- | --- | --- |
| 223961_s_at | cytokine inducible SH2-containing protein | D83532 | 0.029195 | 1154 | CISH | 3p21.3 |
| 1555723_at | SH3 multiple domains 2 | AB062480 | 0.029293 | --- | --- | --- |
| 215633_x_at | leukocyte specific transcript 1 | AV713720 | 0.02937 | 7940 | LST1 | 6p21.3 |
| 238376_at | KIAA0350 protein | AI436581 | 0.029606 | --- | --- | --- |
| 211675_s_at | MyoD family inhibitor domain containing | AF054589 | 0.029623 | 29969 | MDFIC | 7q31.1-q31.2 |
| 224590_at | gb:BE644917 /DB_XREF=gi:9969228 /DB_XREF=7e61e05.x1 /CLONE=IMAGE:3286976 /FEA=mRNA /CNT=416 /TID=Hs.83623.2 /TIER=Stack /STK=21 /UG=Hs.83623 /LL=9970 /UG_GENE=NR1I3 /UG_TITLE=nuclear receptor subfamily 1. group I. member 3 | BE644917 | 0.030076 | 7503 | XIST | Xq13.2 |
| 214181_x_at | leukocyte specific transcript 1 | AI735692 | 0.030217 | 7940 | LST1 | 6p21.3 |
| 244474_at | RAN binding protein 9 | BE549780 | 0.030262 | --- | --- | --- |
| 227802_at | Similar to RUN and FYVE domain-containing 2; Run- and FYVE-domain containing protein | AI075999 | 0.030274 | 22902 | RUFY3 | 4q13.3 |
| 230014_at | Yippee-like 5 (Drosophila) | BF515592 | 0.030509 | --- | --- | --- |
| 243233_at | PABP1-dependent poly A-specific ribonuclease subunit PAN3 | AI701943 | 0.030659 | --- | --- | --- |
| 243046_at | Ubiquitin-conjugating enzyme E2D 3 (UBC4/5 homolog yeast) | BF679700 | 0.030788 | --- | --- | --- |
| 208937_s_at | inhibitor of DNA binding 1. dominant negative helix-loop-helix protein | D13889 | 0.030858 | 3397 | ID1 | 20q11 |
| 210629_x_at | leukocyte specific transcript 1 | AF000425 | 0.031117 | 7940 | LST1 | 6p21.3 |
| 210051_at | Rap guanine nucleotide exchange factor (GEF) 3 | U78168 | 0.031283 | 10411 | RAPGEF3 | 12q13.1 |
| 1556049_at | reticulon 4 | CA428769 | 0.031392 | 57142 | RTN4 | 2p16.3 |
| 240602_at | HBS1-like (S. cerevisiae) | AI801875 | 0.031625 | 10767 | HBS1L | 6q23-q24 |
| 211581_x_at | leukocyte specific transcript 1 | AF000426 | 0.031668 | 7940 | LST1 | 6p21.3 |
| 1554903_at | FKSG44 gene | BC033851 | 0.032083 | 83786 | FRMD8 | 11q13 |
| 232539_at | Suppressor of cytokine signaling 2 | AL161980 | 0.032302 | --- | --- | --- |
| 217110_s_at | mucin 4 tracheobronchial | AJ242547 | 0.032305 | 4585 | MUC4 | 3q29 |
| 201028_s_at | CD99 antigen | U82164 | 0.032571 | 4267 | CD99 | Xp22.32; Yp11.3 |
| 201425_at | aldehyde dehydrogenase 2 family (mitochondrial) | NM_000690 | 0.032819 | 217 | ALDH2 | 12q24.2 |
| 220330_s_at | SAM domain. SH3 domain and nuclear localization signals 1 | NM_022136 | 0.032957 | 64092 | SAMSN1 | 21q11 |
| 204197_s_at | runt-related transcription factor 3 | NM_004350 | 0.033101 | 864 | RUNX3 | 1p36 |
| 229726_at | GRB2-related adaptor protein | AW007479 | 0.033818 | 10750 | GRAP | 17p11.2 |
| 204949_at | intercellular adhesion molecule 3 | NM_002162 | 0.034726 | 3385 | ICAM3 | 19p13.3-p13.2 |
| 210172_at | splicing factor 1 | D26121 | 0.034926 | 7536 | SF1 | 11q13 |
| 237456_at | RING1 and YY1 binding protein | AI655806 | 0.034998 | --- | --- | --- |
| 241620_at | structural maintenance of chromosomes flexible hinge domain containing 1 | AA873021 | 0.035511 | 23347 | SMCHD1 | 18p11.32 |
| 230643_at | Wingless-type MMTV integration site family. member 9A | BE220265 | 0.035523 | 7483 | WNT9A | 1q42 |
| 1558002_at | Serine/threonine kinase receptor associated protein | BQ944989 | 0.035682 | 11171 | STRAP | 12p12.3 |
| 1566959_at | GRB2-associated binding protein 2 | AL049273 | 0.03574 | --- | --- | --- |
| 216920_s_at | T cell receptor gamma constant 2 /// T cell receptor gamma variable 9 /// similar to T-cell receptor gamma chain C region PT-gamma-1/2 /// TCR gamma alternate reading frame protein | M27331 | 0.036467 | 445347 /// 6967 | TARP /// TRGC2 | 7p14 /// 7p15-p14 |
| 230127_at | Transcribed locus | AW044663 | 0.036525 | --- | --- | --- |
| 203234_at | uridine phosphorylase 1 | NM_003364 | 0.036578 | 7378 | UPP1 | 7p12.3 |
| 202124_s_at | amyotrophic lateral sclerosis 2 (juvenile) chromosome region candidate 3 | AV705253 | 0.03675 | 66008 | TRAK2 | 2q33 |
| 205950_s_at | carbonic anhydrase I | NM_001738 | 0.036904 | 759 | CA1 | 8q13-q22.1 |
| 239251_at | Reticulon 4 | AW963634 | 0.036969 | --- | --- | --- |
| 222421_at | ubiquitin-conjugating enzyme E2H (UBC8 homolog. yeast) | BF435617 | 0.037064 | 7328 | UBE2H | 7q32 |
| 221824_s_at | membrane-associated ring finger (C3HC4) 8 | AA770170 | 0.037181 | 220972 | MARCH8 | 10q11.21 |
| 1554501_at | TSC22 domain family 4 | BC031622 | 0.037242 | 81628 | TSC22D4 | 7p21-p15 |
| 225114_at | gb:AA127674 /DB_XREF=gi:1686962 /DB_XREF=zk92b04.s1 /CLONE=IMAGE:490255 /FEA=mRNA /CNT=114 /TID=Hs.331666.0 /TIER=Stack /STK=20 /UG=Hs.331666 /UG_TITLE=Homo sapiens mRNA; cDNA DKFZp762O2215 (from clone DKFZp762O2215) | AA127674 | 0.037281 | 8540 | AGPS | 2q31.2 |
| 204159_at | cyclin-dependent kinase inhibitor 2C (p18. inhibits CDK4) | NM_001262 | 0.037767 | 1031 | CDKN2C | 1p32 |
| 227671_at | gb:AV646597 /DB_XREF=gi:9867611 /DB_XREF=AV646597 /CLONE=GLCAPB04 /FEA=EST /CNT=57 /TID=Hs.51615.0 /TIER=Stack /STK=34 /UG=Hs.51615 /UG_TITLE=ESTs. Weakly similar to ALU7_HUMAN ALU SUBFAMILY SQ SEQUENCE CONTAMINATION WARNING ENTRY (H.sapiens) | AV646597 | 0.038288 | 7503 | XIST | Xq13.2 |
| 213035_at | ankyrin repeat domain 28 | AI081194 | 0.038681 | 23243 | ANKRD28 | 3p24.3 |
| 210275_s_at | zinc finger A20 domain containing 2 | AF062347 | 0.038747 | 7763 | ZFAND5 | 9q13-q21 |
| 208892_s_at | dual specificity phosphatase 6 | BC003143 | 0.03879 | 1848 | DUSP6 | 12q22-q23 |
| 202853_s_at | RYK receptor-like tyrosine kinase | NM_002958 | 0.038921 | 6259 | RYK | 3q22 |
| 1554905_x_at | FKSG44 gene | BC033851 | 0.03917 | 83786 | FRMD8 | 11q13 |
| 244357_at | Hypothetical protein AL133206 | T90760 | 0.0395 | --- | --- | --- |
| 231329_at | Inositol 1.4.5-triphosphate receptor type 1 | N21631 | 0.040122 | --- | --- | --- |
| 213515_x_at | hemoglobin. gamma A /// hemoglobin. gamma A /// hemoglobin. gamma G /// hemoglobin. gamma G | AI133353 | 0.040543 | 3047 /// 3048 | HBG1 /// HBG2 | 11p15.5 |
| 201367_s_at | zinc finger protein 36 C3H type-like 2 | AI356398 | 0.041278 | 678 | ZFP36L2 | 2p22.3-p21 |
| 202672_s_at | activating transcription factor 3 | NM_001674 | 0.04128 | 467 | ATF3 | 1q32.3 |
| 223394_at | SERTA domain containing 1 | BC002670 | 0.041346 | 29950 | SERTAD1 | 19q13.1-q13.2 |
| 216813_at | gb:AL512728.1 /DB_XREF=gi:12224871 /GEN=DKFZp547P082 /FEA=mRNA /CNT=1 /TID=Hs.307068.0 /TIER=ConsEnd /STK=0 /UG=Hs.307068 /DEF=Homo sapiens mRNA; cDNA DKFZp547P082 (from clone DKFZp547P082). /PROD=hypothetical protein | AL512728 | 0.041466 | --- | --- | --- |
| 217762_s_at | RAB31 member RAS oncogene family | BE789881 | 0.041738 | 11031 | RAB31 | 18p11.3 |
| 208893_s_at | dual specificity phosphatase 6 | BC005047 | 0.04188 | 1848 | DUSP6 | 12q22-q23 |
| 243006_at | FYN oncogene related to SRC FGR YES | BG222258 | 0.041999 | --- | --- | --- |
| 201711_x_at | RAN binding protein 2 | AI681120 | 0.042048 | 5903 | RANBP2 | 2q12.3 |
| 212276_at | lipin 1 | D80010 | 0.0424 | 23175 | LPIN1 | 2p25.1 |
| 242836_at | gb:AI800470 /DB_XREF=gi:5365942 /DB_XREF=tj14g11.x1 /CLONE=IMAGE:2141540 /FEA=EST /CNT=5 /TID=Hs.171941.0 /TIER=ConsEnd /STK=3 /UG=Hs.171941 /UG_TITLE=ESTs | AI800470 | 0.042448 | --- | --- | --- |
| 239404_at | Topoisomerase (DNA) I | BF840360 | 0.042521 | --- | --- | --- |
| 235739_at | Nuclear receptor subfamily 4 group A member 2 | AA523939 | 0.04277 | --- | --- | --- |
| 227486_at | 5'-nucleotidase. ecto (CD73) | AI086864 | 0.042888 | 4907 | NT5E | 6q14-q21 |
| 201009_s_at | thioredoxin interacting protein | AI439556 | 0.043805 | 10628 | TXNIP | 1q21.1 |
| 222621_at | DnaJ (Hsp40) homolog. subfamily C. member 1 | BF591419 | 0.043871 | 64215 | DNAJC1 | 10p12.31 |
| 213524_s_at | putative lymphocyte G0/G1 switch gene | NM_015714 | 0.044191 | 50486 | G0S2 | 1q32.2|1q32.2-q41 |
| 218469_at | gremlin 1 homolog. cysteine knot superfamily (Xenopus laevis) | NM_013372 | 0.044375 | 26585 | GREM1 | 15q13-q15 |
| 207735_at | ring finger protein 125 | NM_017831 | 0.044491 | 54941 | RNF125 | 18q12.1 |
| 232685_at | Ets variant gene 6 (TEL oncogene) | AK025217 | 0.044598 | --- | --- | --- |
| 201904_s_at | CTD (carboxy-terminal domain. RNA polymerase IIpolypeptide A) small phosphatase-like | BF031714 | 0.04483 | 10217 | CTDSPL | 3p21.3 |
| 210676_x_at | RAN binding protein 2-like 1 | U64675 | 0.04501 | 727851 /// 729540 /// 84220 | RGPD5 /// RGPD6 /// RGPD8 | 2q13 |
| 232865_at | ALL1 fused gene from 5q31 | N59653 | 0.0453 | 27125 | AFF4 | 5q31 |
| 201008_s_at | thioredoxin interacting protein | AA812232 | 0.045428 | 10628 | TXNIP | 1q21.1 |
| 209582_s_at | CD200 antigen | H23979 | 0.045442 | 4345 | CD200 | 3q12-q13 |
| 228953_at | similar to junction-mediating and regulatory protein p300 JMY | AI023634 | 0.045668 | 123720 | WHDC1 | 15q25.2 |
| 204848_x_at | hemoglobin. gamma A | NM_000559 | 0.045838 | 3047 /// 3048 | HBG1 /// HBG2 | 11p15.5 |
| 204093_at | cyclin H | NM_001239 | 0.046076 | 902 | CCNH | 5q13.3-q14 |
| 60471_at | Ras and Rab interactor 3 | AA625133 | 0.046158 | 79890 | RIN3 | 14q32.12 |
| 204141_at | tubulin beta 2 | NM_001069 | 0.04645 | 7280 | TUBB2A | 6p25 |
| 222620_s_at | DnaJ (Hsp40) homolog subfamily C. member 1 | BF591419 | 0.046794 | 64215 | DNAJC1 | 10p12.31 |
| 242143_at | RAN binding protein 9 | BE674964 | 0.046883 | --- | --- | --- |
| 203290_at | major histocompatibility complex class II DQ alpha 1 | NM_002122 | 0.046899 | 3117 | HLA-DQA1 | 6p21.3 |
| 208891_at | dual specificity phosphatase 6 | BC003143 | 0.047062 | 1848 | DUSP6 | 12q22-q23 |
| 204419_x_at | hemoglobin. gamma A /// hemoglobin. gamma A /// hemoglobin. gamma G /// hemoglobin. gamma G | NM_000184 | 0.047071 | 3047 /// 3048 | HBG1 /// HBG2 | 11p15.5 |
| 224797_at | arrestin domain containing 3 | AB037797 | 0.047771 | 57561 | ARRDC3 | 5q14.3 |
| 1557632_at | CDNA FLJ40660 fis clone THYMU2019686 | BU681135 | 0.048078 | --- | --- | --- |
| 236975_at | Transcribed locus | BF515552 | 0.048165 | --- | --- | --- |
| 236293_at | Ras homolog gene family member H | BE676335 | 0.048415 | --- | --- | --- |
| 226811_at | family with sequence similarity 46. member C | AL046017 | 0.048426 | 54855 | FAM46C | 1p12 |
| 237632_at | Hect (homologous to the E6-AP (UBE3A) carboxyl terminus) domain and RCC1 (CHC1)-like domain (RLD) 1 | AA765387 | 0.048448 | --- | --- | --- |
| 1564077_at | CDNA FLJ23860 fis clone LNG08308 | AK074440 | 0.048573 | --- | --- | --- |
| 209301_at | carbonic anhydrase II | M36532 | 0.048684 | 760 | CA2 | 8q22 |
| 211796_s_at | T cell receptor beta constant 1 | AF043179 | 0.048892 | 28638 /// 28639 | TRBC1 /// TRBC2 | 7q34 |
| 233379_at | Hypothetical protein FLJ14213 | AK026283 | 0.049117 | 79899 | FLJ14213 | 11p13-p12 |
| 201200_at | cellular repressor of E1A-stimulated genes 1 | NM_003851 | 0.049781 | 8804 | CREG1 | 1q24 |
