# Supplementary material for: IKAROS Deletions Dictate a Unique Gene Expression Signature in Patients with Adult B-Cell Acute Lymphoblastic Leukemia
Source: PLoS One. 2012 Jul 25;7(7):e40934. doi: 10.1371/journal.pone.0040934 (PMC3405023; doi:10.1371/journal.pone.0040934)
Supplement: Table S4 — Treatment outcome and results of therapy related to IKZF1 loss in univariate analysis. Abbreviations: mths (months), wt (wild-type). (DOCX) [file pone.0040934.s007.docx]

|  | | ***IKZF1 status*** | | | |
| --- | --- | --- | --- | --- | --- |
|  |  | **ALL Patients**  **% (C.I.95%)** | ***IKZF1* wt**  **% (C.I.95%)** | ***IKZF1* loss**  **% (C.I.95%)** | **p-value** |
| Cumulative Incidence  of Relapse  (CIR) | **Patients** | **121** | **46** | **75** | 0.02 |
|  | at 24 mths | 55.4% (54.9-55.9) | 44% (42.5-45.5) | 63.3% (62.5-64) |  |
|  | Median time | 16.4 mths | 45 mths | 11.6 mths |  |
| Disease free survival (DFS) | **Patients** | **121** | **46** | **75** | 0.04 |
|  | at 24 mths | 42.5% (38.6-46.9) | 50.8% (42.9-60.2) | 49.7% (47.5-51.8) |  |
|  | Median time | 16.3 mths | 32.1 mths | 11.6 mths |  |
