# Supplementary material for: IKAROS Deletions Dictate a Unique Gene Expression Signature in Patients with Adult B-Cell Acute Lymphoblastic Leukemia
Source: PLoS One. 2012 Jul 25;7(7):e40934. doi: 10.1371/journal.pone.0040934 (PMC3405023; doi:10.1371/journal.pone.0040934)
Supplement: Table S5 — IKZF1 deletion and other clinical relevant factors for predicting Disease Free Survival (multivariate analysis). Abbreviations: WBC (white blood cells). (DOCX) [file pone.0040934.s008.docx]

|  | **Hazard ratio** | **95% CI** | **p-value** |
| --- | --- | --- | --- |
| ***IKZF1* deletion** |  |  |  |
| Yes vs No | 1.665 | 0.960-5.523 | 0.0696 |
| **Protocol treatment** |  |  |  |
| LAL 1205 vs. LAL 2000 | 1.852 | 0.621-13.443 | 0.2689 |
| LAL 0201-B vs. LAL 2000 | 4.284 | 1.365-9.341 | 0.0126 |
| LAL 0904 vs. LAL 2000 | 3.406 | 1.242-6.551 | 0.0173 |
| Institutional protocols vs. LAL 2000 | 1.937 | 0.573-2.887 | 0.2874 |
| ***BCR-ABL1*:** |  |  |  |
| Positive vs. negative | 1.008 | 0.303-3.356 | 0.0026 |
| **WBC at diagnosis (as continuous variable)** | 1.006 | 1.002-1.010 | 0.9893 |
