# Supplementary material for: IKAROS Deletions Dictate a Unique Gene Expression Signature in Patients with Adult B-Cell Acute Lymphoblastic Leukemia
Source: PLoS One. 2012 Jul 25;7(7):e40934. doi: 10.1371/journal.pone.0040934 (PMC3405023; doi:10.1371/journal.pone.0040934)
Supplement: Table S6 — Description of GIMEMA clinical trials enrolling patients who were analyzed in this study. (DOC) [file pone.0040934.s009.doc]

| **Name** | **Clinical Trial Gov Identifier** | **Eligibility** | **Inclusion Criteria** | **References** |
| --- | --- | --- | --- | --- |
| **GIMEMA Guidelines for the Treatment of Adult ALL Affected Patients at Diagnosis**  **(LAL 2000)** | NCT00537550 | - 14 Years to 60 Years - Both genders - No Healthy Volunteers | - Diagnosis of ALL - Negative myeloperoxidase stain - Phenotype T (ALL-T) and B (ALL-B) | [1] |
| **Combination Chemotherapy With or Without Imatinib Mesylate and/or Peripheral Stem Cell Transplant in Treating Patients With Acute Lymphoblastic Leukemia (LAL 0904)** | NCT00458848 | - 15 Years to 60 Years - Both genders - No Healthy Volunteers | - DISEASE CHARACTERISTICS: Diagnosis of ALL meeting the following criteria: Negative myeloperoxidase stain; Phenotype T (T-ALL) or B (B-ALL); No mature B-ALL (FAB L3, serum immunoglobulin-positive, terminal deoxynucleotidyl transferase-negative). - PATIENT CHARACTERISTICS: Creatinine ≤ 2.5 mg/dL (after adequate hydration); SGOT and SGPT ≤ 3 times upper limit of normal; LVEF ≥ 50%; No severe psychiatric disorders; No other concurrent malignant disease; No presence of documented infections not responding to antibiotic and/or antifungal therapy; Not pregnant. - PRIOR CONCURRENT THERAPY: No prior steroids; No prior antiblastic chemotherapy; No other concurrent chemotherapy or radiotherapy. | [1] |
| **A Phase II Multicenter Study on the Treatment of Adult de Novo Philadelphia Chromosome Positive (Ph+) Acute Lymphoblastic Leukemia (ALL) With the Protein Tyrosine Kinase Inhibitor BMS-354825 (LAL 1205)** | NCT00391989 | - 18 Years or older - Both genders - No Healthy Volunteers | - Patients with Ph+ and/or BCR/ABL+ ALL - Age ≥18 years old - De novo ALL (within 14 days from diagnosis) - No prior treatment with any anti-leukemic drugs with the exception of steroids for no more than 14 days (including the 7-day pretreatment already scheduled in the protocol) - WHO performance status ≤2 - Absence of central nervous system (CNS) leukemia - Normal serum level of potassium, total calcium corrected for serum albumin magnesium and phosphorus, or correctable with supplements - ALT and AST ≤2.5 x ULN or ≤5.0 x ULN if considered due to leukemia - Alkaline phosphatase ≤2.5 x ULN unless considered to leukemia - Serum bilirubin ≤2 x ULN - Serum creatinine ≤3 x ULN - Serum amylase ≤1.5 x ULN and serum lipase ≤1.5 x ULN - Normal cardiac function - Written informed consent prior to any study procedures being performed. | [2] |
| **STI 571 (GLIVEC) in the Treatment of Philadelphia-chromosome Positive and/or BCR/ABL Rearranged Adult Acute Lymphoblastic Leukemia (LAL 0201)** | NCT00376467 | - 18 Years or older - Both genders - No Healthy Volunteers | - Patients with Ph +ve and/or BCR/ABL +ve ALL, either in 1st CHR (independently from the molecular status) for study A, or at diagnosis and untreated for study B; - Age >18 years and <60 for study A, >60 for study B; - Written voluntary informed consent. | [3] |

References of the table:

1. Cimino G, Cenfra N, Elia L, Sica S, Luppi M, et al. (2010) The therapeutic response and clinical outcome of adults with ALL1(MLL)/AF4 fusion positive acute lymphoblastic leukemia according to the GIMEMA experience. Haematologica 95: 837-840.

2. Foa R, Vitale A, Vignetti M, Meloni G, Guarini A, et al. (2011) Dasatinib as first-line treatment for adult patients with Philadelphia chromosome-positive acute lymphoblastic leukemia. Blood 118: 6521-6528.

3. Vignetti M, Fazi P, Cimino G, Martinelli G, Di Raimondo F, et al. (2007) Imatinib plus steroids induces complete remissions and prolonged survival in elderly Philadelphia chromosome-positive patients with acute lymphoblastic leukemia without additional chemotherapy: results of the Gruppo Italiano Malattie Ematologiche dell'Adulto (GIMEMA) LAL0201-B protocol. Blood 109: 3676-3678.
