# Supplementary material for: IKAROS Deletions Dictate a Unique Gene Expression Signature in Patients with Adult B-Cell Acute Lymphoblastic Leukemia
Source: PLoS One. 2012 Jul 25;7(7):e40934. doi: 10.1371/journal.pone.0040934 (PMC3405023; doi:10.1371/journal.pone.0040934)
Supplement: Table S7 — Specific pairs of primers used for quantitative ChIP analysis. Different amplicons were analyzed for each gene. Abbreviations: F (forward), R (reverse). (DOCX) [file pone.0040934.s010.docx]

| **Primer name** | **Sequence 5’→3’** |
| --- | --- |
| *IGLL1* amplicon A F | CCCACAAAATAATGCCACAGAAAC |
| *IGLL1* amplicon A R | CCTTGGACATGAGGATGATGATG |
| *IGLL1* amplicon B F | CCCATCCATAGGAGGTATCCAGAG |
| *IGLL1* amplicon B R | CCATTCAACACCAACTTCCCATTC |
| *IGLL1* amplicon C F | GGGGAAGTGGGGAAGGAGGGAAGTC |
| *IGLL1* amplicon C R | TGAGGTCACACAGGGAGGTGGGATG |
| *IGLL1* amplicon D F | GAGGGGTGAGGAGGAAGGTTAATC |
| *IGLL1* amplicon D R | GGGAGGTGAGGGAGACATTGG |
| *BLK* amplicon A F | TGATGAGAAGTCAACCACCAATC |
| *BLK* amplicon A R | AGAATCACACCTAGATGTCATAGTC |
| *BLK* amplicon B F | GCCCACCATTCATACCCATTCTAC |
| *BLK* amplicon B R | CAGCCGTTCTTTCAGCCTCAC |
| *BLK* amplicon C F | CCTCGGGAGAGTGGGACTTAATTC |
| *BLK* amplicon C R | CTGTCGTGGCAGTTCACAATAGC |
| *EBF1* amplicon A F | CTAAGGTAATTGTGATGGATAGTGC |
| *EBF1* amplicon A R | TTCTAAATCTTCATGTGAGGGTAGC |
| *EBF1* amplicon B F | GGGTTAGTGTGCCTGTGTTTAG |
| *EBF1* amplicon B R | CCCTGCTGGATGGAGATTCTG |
| *EBF1* amplicon C F | CCCAACAGCCAAGCATACATC |
| *EBF1* amplicon C R | GAACTCGCACTTAGAAGATCAAGG |
| *BUB3* amplicon A F | GTCCAAGGAGTCGGAGTTGC |
| *BUB3* amplicon A R | CATCAGCGGCGTCTAACCAG |
| *BUB3* amplicon B F | CGGAACAGGATGACTGGGTTGAC |
| *BUB3* amplicon B R | TAGGCGAAGGAGGCGGAGAG |
| *MSH2* amplicon A F | GGCTGATTAGACCCTGAGAAC |
| *MSH2* amplicon A R | GGTGTGACTGAATATGTTAATGAG |
| *MSH2* amplicon B F | AGACTCCCACCCACCGAAAC |
| *MSH2* amplicon B R | CGAAACCTCCTCACCTCCTG |
| *MCL1* amplicon A F | TCACATCTGTAATCCCAGCACTTTG |
| *MCL1* amplicon A R | CCTCCTGAGTTCAAGCCATTATCC |
| *MCL1* amplicon B F | TCTTCCTTCAACCCCTGTGTTAGTC |
| *MCL1* amplicon B R | CTTCCCTGAGACCTGATTTGTGAGC |
| *MCL1* amplicon C F | GCCGCCCTAAAACCGTGATAAAG |
| *MCL1* amplicon C R | CACAGTAGAGGTTGAGTCCGATTAC |
| *ETV6* amplicon A F | GCTGGGCGTGGTTGTAGG |
| *ETV6* amplicon A R | TTTGAAATGGAGTCTTGCTCTGTC |
| *ETV6* amplicon B F | GGGGAGGTCAGGAAAAGGAAC |
| *ETV6* amplicon B R | GGAAATGGGAGATCGGGTTACAG |
| *ETV6* amplicon C F | GGAGCCTTTCTGGGTTGG |
| *ETV6* amplicon C R | CTACACTGAGCAGGAGTCTC |
| *YES1* amplicon A F | ATGTGGTTTCAAGTATTCAG |
| *YES1* amplicon A R | ATCATATTCATTTCTTTATAGTGG |
| *YES1* amplicon B F | GGCGGAGGAGGTGGAGAGTG |
| *YES1* amplicon B R | GGCAGCAACGACGGCAGAG |
| *CDKN1A* amplicon A F | TTCTCCTGACAGAGTGATTATCG |
| *CDKN1A* amplicon A R | CTATCAATGGGTTTGGGTTATCG |
| *CDKN1A* amplicon B F | TGCCGAAGTCAGTTCCTTGTGG |
| *CDKN1A* amplicon B R | GCCGCTCTCTCACCTCCTCTG |
| *CDKN2C* amplicon A F | TGTCTCAGTAGCCTCTTCCAATC |
| *CDKN2C* amplicon A R | CCTCTGCGGTGATACACATCTC |
| *CDKN2C* amplicon B F | CTCAAGCCCGCCAGCAGAAG |
| *CDKN2C* amplicon B R | TCCACCTCCTCCCGTCAAGTC |
| *MSH6* amplicon A F | CTTTGCTGGCTGAAATTATTG |
| *MSH6* amplicon A R | CTACTGGCGATGTTGTGGAAGA |
| *MSH6* amplicon B F | CAGAGAGGCAGGGCTTTCCG |
| *MSH6* amplicon B R | CTGGCACACTGGTGGGTAGG |
| *BTK* amplicon A F | GCCTTGATTGCTCCTTTTACC |
| *BTK* amplicon A R | CAGTGTTGGTTTGTTAATGAGTC |
| *BTK* amplicon B F | AAGGGTGGGGTTTGCTCAGAC |
| *BTK* amplicon B R | CAGGACTTGGAAGGTGGGACTC |
| *VPREB1* amplicon A F | CAGAACAAGAGGCTAAAGAGTG |
| *VPREB1* amplicon A R | AACGAACGAATGAAGGAATGC |
| *VPREB1* amplicon B F | TACTTGGTTCTAGGAGGGAGAGGAG |
| *VPREB1* amplicon B R | GCCAGGAAGGGAGGACACATC |
| *VPREB1* amplicon C F | CTGTCCTGCTCATGCTGTTTGTC |
| *VPREB1* amplicon C R | AGGTGAGGCGGATTGTGGTTC |
| *CD22* amplicon A F | ATATGTAAGAGTGGAGATGC |
| *CD22* amplicon A R | CTGGAAAGATGTATGGAAAC |
| *CD22* amplicon B F | AAACCCATACACGGAAAGGAAGAAC |
| *CD22* amplicon B R | CCCTGGCAGCATCTGAGAGC |
