# Supplementary material for: IKAROS Deletions Dictate a Unique Gene Expression Signature in Patients with Adult B-Cell Acute Lymphoblastic Leukemia
Source: PLoS One. 2012 Jul 25;7(7):e40934. doi: 10.1371/journal.pone.0040934 (PMC3405023; doi:10.1371/journal.pone.0040934)
Supplement: Appendix S1 — Authors of GIMEMA contributing to the study. (DOCX) [file pone.0040934.s011.docx]

**Appendix S1**

**Authors of GIMEMA contributing to the study:** LEONE Giuseppe - Università Cattolica del Sacro Cuore - Policlinico A. Gemelli, Rome; TORELLI Giuseppe - Centro Oncologico Modenese - Dipartimento di Oncoematologia, Modena; FERRARA Felicetto - Azienda Ospedaliera di Rilievo Nazionale "A. Cardarelli", Napoli; MAJOLINO Ignazio - Divisione di Ematologia - Ospedale S. Camillo, Rome; FANIN Renato - Clinica Ematologica, Policlinico Universitario, Udine; PIZZOLO Giovanni e BONIFACIO Massimiliano - Università degli Studi di Verona - A. O. - Istituti Ospitalieri di Verona- Div. di Ematologia – Policlinico G.B. Rossi, Verona; DI RAIMONDO Francesco - Università di Catania - Cattedra di Ematologia - Ospedale "Ferrarotto" – Catania; MORRA Enrica - Ospedale Niguarda "Ca Granda" – Milano; MIRTO Salvatore - Div. di Ematologia - A.O. "V. Cervello" – Palermo; NOBILE Francesco - Dipartimento Emato-Oncologia A.O."Bianchi-Melacrino-Morelli" - Reggio Calabria; LONGINOTTI Maurizio - Serv. di Ematologia Ist. di Ematologia ed Endocrinologia – Sassari; QUARTA Giovanni - Divisione di Ematologia Osp. Reg. A. Di Summa – Brindisi; LISO Vincenzo - Unità Operativa Ematologia 1 - Università degli Studi di Bari - Padiglione Chini -3°piano – Bari; PETA Antonio - Azienda Ospedaliera Pugliese Ciaccio - Presidio Ospedaliero A.Pugliese - Unità Operativa di Ematologia – Catanzaro; ROTOLI Bruno - Azienda Ospedaliera Universitaria - Università degli Studi di Napoli "Federico II" Facoltà di Medicina, Napoli; FIORITONI Giuseppe - U.O. Ematologia Clinica - Azienda USL di Pescara; OLIVIERI Attilio - Ematologia - Ospedale San Carlo – Potenza; DE FABRITIIS Paolo - U.O.C. Ematologia - Ospedale S.Eugenio – Roma; BOCCADORO Mario - Div. di Ematologia Ospedale "S.Giovanni Battista" – Torino; SAGLIO Giuseppe - Dip. di Scienze Cliniche e Biologiche - Ospedale S. Luigi Gonzaga - Orbassano (TO); MONTANARO Marco - Azienda Sanitaria Locale Viterbo - Polo Ospedaliero Centrale - Ospedale Di Ronciglione - U.O. di Ematologia - Ronciglione (Viterbo); ZACCARIA Alfonso - Dipartimento Oncologico - Ospedale S.Maria delle Croci – Ravenna; D'ARCO Alfonso Maria - U.O. Medicina Interna Ematologia ed Oncologia P.O. Umberto I - Nocera Inferiore (SA); BRUGIATELLI Maura - Divisione di Ematologia - Azienda Ospedaliera "Papardo" – Messina; GAIDANO Gianluca - S.C.D.U. Ematologia - DIMECS e Dipartimento Oncologico - Università del Piemonte Orientale Amedeo Avogadro – Novara; AMADORI Sergio - Università degli Studi - Policlinico di Tor Vergata – Roma.
